# Pharmacological HIF-1 activation upregulates extracellular vesicle production synergistically with adiponectin through transcriptional induction and protein stabilization of T-cadherin

**DOI:** 10.1038/s41598-024-51935-6

**Published:** 2024-02-13

**Authors:** Kohei Fujii, Yuya Fujishima, Shunbun Kita, Keitaro Kawada, Keita Fukuoka, Taka-aki Sakaue, Tomonori Okita, Emi Kawada-Horitani, Hirofumi Nagao, Shiro Fukuda, Norikazu Maeda, Hitoshi Nishizawa, Iichiro Shimomura

**Affiliations:** 1 Department of Metabolic Medicine, Graduate School of Medicine, Osaka University, 2-2, Yamada-oka, Suita, Osaka 565-0871 Japan; 2 Department of Adipose Management, Graduate School of Medicine, Osaka University, 2-2, Yamada-oka, Suita, Osaka 565-0871 Japan; 3 Department of Metabolism and Atherosclerosis, Graduate School of Medicine, Osaka University, 2-2, Yamada-oka, Suita, Osaka 565-0871 Japan; 4 Department of Endocrinology, Metabolism and Diabetes, Faculty of Medicine, Kindai University, 377-2, Ohno-higashi, Osaka-Sayama, Osaka 589-8511 Japan

**Keywords:** Cell biology, Molecular biology

## Abstract

Pharmacological activation of hypoxia-inducible factor 1 (HIF-1), a hypoxia-responsive transcription factor, has attracted increasing attention due to its efficacy not only in renal anemia but also in various disease models. Our study demonstrated that a HIF-1 activator enhanced extracellular vesicle (EV) production from cultured endothelial cells synergistically with adiponectin, an adipocyte-derived factor, through both transcriptional induction and posttranscriptional stabilization of an adiponectin binding partner, T-cadherin. Increased EV levels were observed in wild-type mice but not in T-cadherin null mice after consecutive administration of roxadustat. Adiponectin- and T-cadherin-dependent increased EV production may be involved in the pleiotropic effects of HIF-1 activators.

## Introduction

Intracellularly synthesized nanoscale extracellular vesicles (EVs), called exosomes, contribute to whole-body homeostasis by removing unwanted substances and/or transducing intercellular signals^1^. T-cadherin, a glycosylphosphatidylinositol (GPI)-anchored binding partner of adiponectin, mediates EV biogenesis triggered by adiponectin, an adipocyte-derived secreted factor known to have several organ-protective functions^2^.

Recently, we reported that native hexameric or larger adiponectin specifically binds to cell surface T-cadherin with very high affinity, is endocytosed into endosomes, and increases EV biogenesis from cells expressing T-cadherin, such as endothelial cells^3,4^. This function has been implicated in ceramide efflux from endothelial cells and in the reduction of aortic ceramide accumulation in angiotensin II-induced hypertension^4^. The adiponectin/T-cadherin system has also been associated with improved muscle regeneration and amelioration of renal tubular damage after ischemia–reperfusion injury^5,6^. Applying our findings to mesenchymal stem cell (MSC) therapy, the cell-therapeutic effect of MSCs in a pressure-overloaded heart failure model was found to depend on MSC T-cadherin expression and EV secretion, as well as adiponectin levels in recipient mice^7^. The importance of MSC-derived EVs was also noted in the immune checkpoint inhibitor-associated autoimmune diabetes in a nonobese diabetic (NOD) mouse model^8^. Given such pleiotropic organ protection of adiponectin, the current strategy of increasing T-cadherin expression and its EV-producing function has therapeutic potential for various diseases^9^.

**Figure 1:**
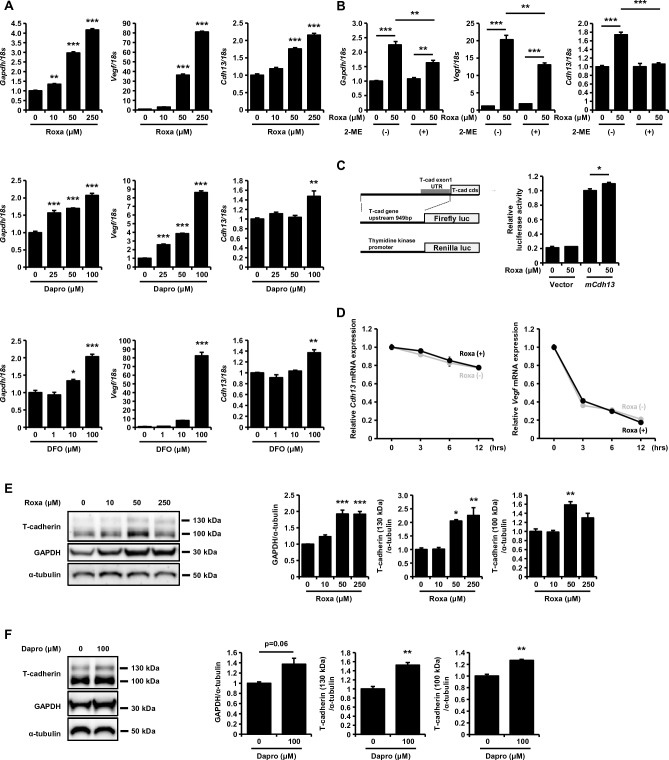
HIF-1 activation upregulated transcription of the T-cadherin mRNA and increased T-cadherin protein in UV-F2 endothelial cells. (**A**) Quantitative PCR analysis of murine endothelial UV-F2 cells treated with roxadustat (Roxa) for 24 h, daprodustat (Dapro) for 48 h, or deferoxamine (DFO) for 24 h at the indicated concentrations (n = 3 for each group). Data are means ± SEMs. *p < 0.05, **p < 0.01, and ***p < 0.001 versus control (Dunnett's test). (**B**) Quantitative PCR analysis of UV-F2 cells treated with or without roxadustat (50 μM) and 1 μM of 2-methoxyestradiol (2-ME), an inhibitor of HIF-1, for 24 h (n = 3 for each group). Data are means ± SEMs. *p < 0.05, **p < 0.01, and ***p < 0.001 (Tukey–Kramer test). (**C**) The constructs used for reporter assay were summarized (left). Luciferase reporter assay using the promoter region of the mouse T-cadherin gene (*mCdh13*) in HEK293 cells. Luciferase activities 24 h after treatment with or without roxadustat (50 μM) were quantified by calculating Firefly luciferase activity against an internal standard, Renilla luciferase activity (n = 3 for each group). Data are means ± SEMs. *p < 0.05 (unpaired t test). (**D**) mRNA stability assay using actinomycin D. UV-F2 cells were incubated with or without roxadustat (50 μM) and 5 μg/mL of actinomycin D for the time indicated (n = 3 for each group). (**E**) and (**F**) Western blot analysis of total cell lysates. UV-F2 cells were treated with roxadustat (**E**) or daprodustat (**F**) for 48 h at the indicated concentrations (n = 3 for each group). Data are means ± SEMs. *p < 0.05, **p < 0.01, and ***p < 0.001 versus control (Dunnett's test or unpaired t test).

By searching for the transactivation factor of T-cadherin in its genomic region, we found two conserved potential hypoxia-inducible factor (HIF)-1 responsive elements in the upstream region of the T-cadherin gene (Supple. Fig. 1A). HIF is a key regulator of the body's response to hypoxia. An oxygen-sensitive enzyme, prolyl hydroxylase (PHD), catalyzes the degradation of the HIF-1α subunit under normoxic conditions. Under hypoxic conditions, however, PHD activity is inhibited, resulting in the stabilization and transactivation of HIF-1 responsive genes, such as *Vegf*^10,11^. Previous studies have reported that hypoxic conditions in the tumor microenvironment stimulate the generation and secretion of EVs from cancer cells, which can trigger cancer-promoting effects such as angiogenesis, invasion, and metastasis^12,13^. Pharmacological activators of HIF-1 targeting PHD are approved for clinical use in the treatment of renal anemia in patients with chronic kidney disease (CKD)^11^. A recent placebo-controlled randomized phase 3 study of roxadustat, a selective HIF-PHD inhibitor, in 2781 patients with CKD showed no increased risk of serious adverse events, including cancer^14^. Interestingly, these HIF-1 activators have been reported to have potential not only in anemia but also in various disease models^10,11,15,16^.

Here, we demonstrated that pharmacological HIF-1 activation induced T-cadherin expression through both transactivation and posttranscriptional protein stabilization by downregulating a juxta-membrane sheddase of a disintegrin and metalloproteinase 12 (ADAM12), leading to enhanced EV production by purified high-molecular weight multimer adiponectin. Three consecutive daily doses of roxadustat, a selective HIF-PHD inhibitor, increased the number of plasma EVs in wild-type but not in T-cadherin null mice.

## Results

### Pharmacological HIF-1 activation increased both T-cadherin mRNA and protein levels in endothelial cells

By searching for potential transcription factors regulating T-cadherin expression, we found two typical hypoxia response element (HRE) sites upstream of the T-cadherin open reading frame (ORF) in both human and mouse genomes (Supple. Fig. 1A). We tested whether HIF-1 activation can increase T-cadherin expression by treating the murine endothelial cell line UV-F2 with the HIF-1 activators, roxadustat, dapurodustat, and deferoxamine (Fig. 1A). Although the T-cadherin gene, *Cdh13*, is not a superior target to the known HIF-1 responsive genes, *Gapdh* and *Vegf*, all these HIF-1 activators significantly increased *Cdh13* mRNA in cultured endothelial cells. Roxadustat gradually increased the T-cadherin mRNA during 48 h of incubation (Supple Fig. 1B). Such T-cadherin gene induction by roxadustat was attenuated by 2-methoxyestradiol, an inhibitor of HIF-1, as were the other HIF response genes, *Vegf* and *Gapdh* (Fig. 1B), and was also observed in other T-cadherin-expressing cells, such as cultured mesenchymal stem cells (Supple. Fig. 1C). Luciferase reporter assay in HEK293 cells showed that the upstream region of the mouse T-cadherin gene containing the two HRE sites had substantial promoter activity and was significantly activated by roxadustat (Fig. 1C). In contrast, T-cadherin mRNA degradation, as assessed by transcriptional inhibition with actinomycin D treatment of UV-F2 cells, was not altered by roxadustat-induced HIF activation and was more stable compared to *Vegf* mRNA (Fig. 1D).

The protein levels of T-cadherin and GAPDH were also significantly increased by treatment with roxadustat or daprodustat in UV-F2 cells (Fig. 1E and F), with the 130 kDa form of T-cadherin being more prevalent than the 100 kDa form. T-cadherin mRNA (Supple. Fig. 1D) and protein (Supple. Fig. 1E) upregulation by roxadustat was also observed in human umbilical vein endothelial cells (HUVECs). Although the dose-dependence was not straightforward (Fig. 1E and Supple. Fig. 1E), the highest dose of roxadustat accompanied the increase in *Chop* gene expression (Supple. Fig. 1F), a marker of endoplasmic reticulum (ER) stress, which could be associated with downregulation of T-cadherin, as we reported previously^17^. Hypoxia conditions (5% O_2_) did not alter *Cdh13* gene expression (Supple. Fig. 1G) or T-cadherin protein levels (Supple. Fig. 1H). These results indicate that selective HIF-1 activation increases T-cadherin expression, in part through its transcriptional regulation.

### HIF-1 activation increases the cellular accumulation of adiponectin and adiponectin-dependent EV production in a T-cadherin-dependent manner

**Figure 2:**
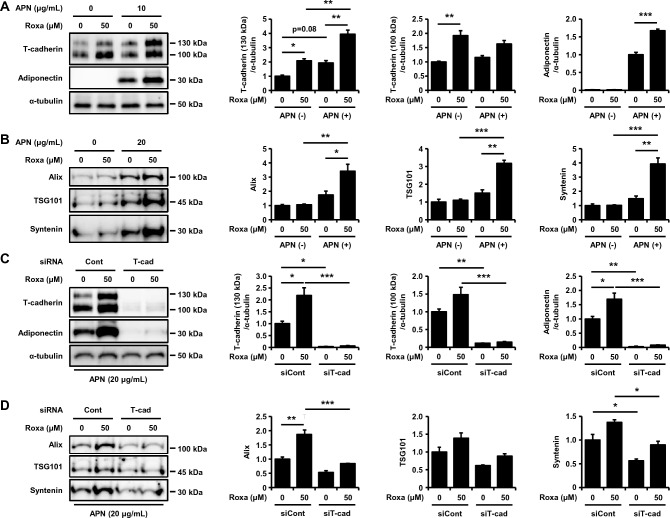
HIF-1 activation-induced T-cadherin upregulation increased adiponectin accumulation and EV production. (**A**) Western blot analysis of total cell lysates. UV-F2 cells cultured in DMEM containing 5% serum from adiponectin knockout mice were treated with or without high molecular weight adiponectin (HMW-APN) (10 μg/mL) and roxadustat (Roxa) (50 μM) for 48 h (n = 3 for each group). (**B**) Western blot analysis of EVs isolated from cell culture medium by differential ultracentrifugation. UV-F2 cells cultured in FBS-free Advanced DMEM were treated with or without HMW-APN (20 μg/mL) and roxadustat (50 μM) for 48 h (n = 3 for each group). Alix, TSG101, and syntenin were evaluated as EV markers. (**C**) Western blot analysis of total cell lysates. UV-F2 cells transfected control (Cont) or T-cadherin (T-cad) siRNA were cultured in DMEM containing 5% serum from adiponectin knockout mice with or without HMW-APN (20 μg/mL) and roxadustat (50 μM) for 48 h (n = 3 for each group). (**D**) Western blot analysis of EVs isolated from cell culture medium. UV-F2 cells transfected Cont or T-cad siRNA were cultured in FBS-free Advanced DMEM with or without HMW-APN (20 μg/mL) and roxadustat (50 μM) for 48 h (n = 3). Data are means ± SEMs. *p < 0.05, **p < 0.01, and ***p < 0.001 (Tukey–Kramer test).

Next, we tested whether pharmacological activation of HIF-1 increases adiponectin-dependent EV production. Therefore, we treated UV-F2 cells with roxadustat in the presence or absence of high molecular weight (HMW) adiponectin (Fig. 2A). Roxadustat repeatedly increased T-cadherin protein levels. Importantly, T-cadherin protein levels in the cells were synergistically increased by adiponectin and roxadustat, which was accompanied by a synergistic increase in adiponectin accumulation in the cells (Fig. 2A). The EV amounts evaluated with EV-specific marker protein levels, apoptosis-linked gene 2-interacting protein X (Alix), tumor susceptibility gene (TSG101), and syntenin, in the purified EV fractions were also synergistically increased by adiponectin and roxadustat (Fig. 2B). Importantly, roxadustat treatment itself did not show significant increase in EV production. The effect was only evident in the presence of the physiological concentration of adiponectin used in this study (Fig. 2B). Because adiponectin-dependent EV production requires the cellular expression of T-cadherin in all cells examined thus far^4–7^, we tested whether such a synergistic increase in EV production also requires T-cadherin expression in cells by siRNA-mediated T-cadherin knockdown experiments (Fig. 2C and D). T-cadherin knockdown readily depleted T-cadherin protein expression with concomitant depletion of the cellular accumulation of adiponectin (Fig. 2C). Under these conditions, roxadustat did not increase EV production in the presence of adiponectin (Fig. 2D). Therefore, it is suggested that the EV enhancing effects of HIF-1 activators require both adiponectin and cellular T-cadherin expression.

### HIF-1 activation stabilizes T-cadherin protein in cultured endothelial cells

**Figure 3:**
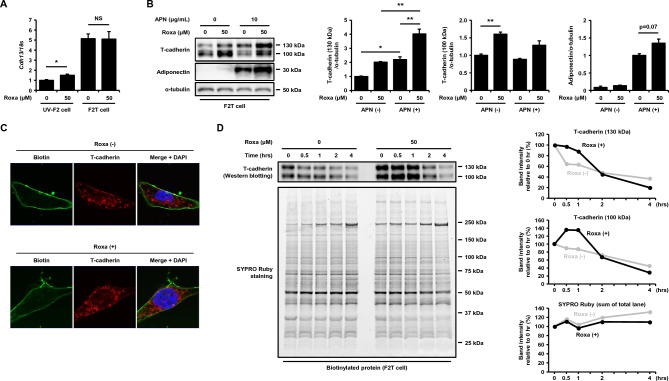
HIF-1 activation posttranscriptionally increased T-cadherin protein levels. (**A**) Quantitative PCR analysis of T-cadherin mRNA. UV-F2 cells and those stably overexpressing T-cadherin (F2T cells) were treated with or without roxadustat (Roxa) (50 μM) for 24 h (n = 3 for each group). Data are means ± SEMs. *p < 0.05 (unpaired t test). NS; not significant. (**B**) Western blot analysis of total cell lysates. F2T cells cultured in DMEM containing 5% serum from adiponectin knockout mice were treated with or without high molecular weight adiponectin (HMW-APN) (10 μg/mL) and roxadustat (50 μM) for 48 h (n = 3 for each group). Data are means ± SEMs. *p < 0.05 and **p < 0.01 (Tukey–Kramer test). (C) and (D) In the presence of HMW-APN (10 μg/mL), F2T cells treated with or without roxadustat (50 μM) for 40 h were biotinylated on the cell surface for 30 min at 4 ℃. (**C**) Representative immunofluorescence images for biotin (*green*) and T-cadherin (*red*) after cell-surface biotinylation. Scale bar = 20 μm. (**D**) The biotinylated cells on the cell surface were further incubated with or without roxadustat for the time indicated. Western blot analysis of T-cadherin and SYPRO Ruby (total protein) in the cell lysates immunoprecipitated with Streptavidin Sepharose™ beads at 0, 0.5, 1, 2, and 4 h after biotinylation. The right panels plot percent changes in band intensities of 130 kDa (top) and 100 kDa (middle) T-cadherin and the sum of the SYPRO Ruby staining (bottom) relative to 0 h at the time indicated for each group (with or without roxadustat).

T-cadherin is expressed at different levels in various cell types, including endothelial cells^18^. The UV-F2 cells that we used in this study expressed far fewer T-cadherin than intact endothelial cells in vivo^4^. In addition, adiponectin increases T-cadherin protein levels by stabilizing T-cadherin as we previously reported^19^. To investigate additional mechanisms underlying the roxadustat-induced synergistic enhancement of T-cadherin protein with adiponectin, we used UV-F2 cells stably overexpressing T-cadherin (F2T cells)^4^. The retrovirally transduced full-length mouse T-cadherin construct was out of HRE-specific regulation and roxadustat did not alter T-cadherin mRNA levels in F2T cells (Fig. 3A). Interestingly, T-cadherin protein was still increased by roxadustat treatments in F2T cells, and roxadustat further increased T-cadherin protein levels synergistically with adiponectin (Fig. 3B). These results suggested that roxadustat increased T-cadherin protein posttranscriptionally. Then, we evaluated the protein stability of T-cadherin located on the cell surface over time by surface biotinylation experiments. Immunofluorescence staining showed that cell surface biotinylation after 40 h incubation with or without roxadustat resulted in clear cell surface labeling with biotin (*green*), and T-cadherin (*red*) staining was stronger with roxadustat on both the cell surface and intracellular structures (Fig. 3C). Western blotting also showed that the biotinylated T-cadherin was increased at the beginning of biotinylation (0 h) in the presence of roxadustat compared to that in the absence of roxadustat (Fig. 3D, T-cadherin). During the chase period of biotinylated protein, biotinylated T-cadherin gradually disappeared. As shown in Fig. 3D, in the top and middle of the right panels, both the 130 kDa and 100 kDa T-cadherin band intensities, relative to those at the beginning of the chase (0 h), were more retained in the presence of roxadustat at 0.5 and 1 h than in the absence of roxadustat. Under these conditions, the total amount of biotinylated protein present on the cell surface at the beginning of biotinylation (0 h) did not change with or without roxadustat (Fig. 3D, SYPRO Ruby), nor did its rate of disappearance differ between the presence and absence of roxadustat (Fig. 3D, bottom of the right panels). These results indicate that, among cell membrane proteins, the clearance of T-cadherin was rapid and stabilized by selective HIF-1 activation.

### HIF-1 activation decreases ADAM12 and thereby stabilizes the T-cadherin protein in cultured endothelial cells

**Figure 4:**
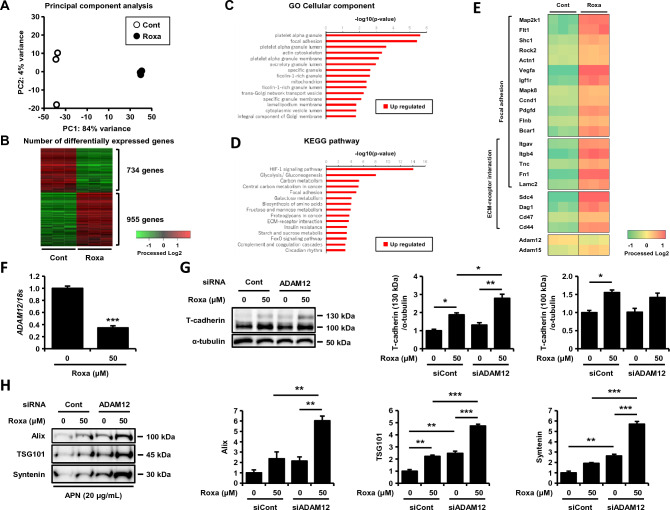
T-cadherin was stabilized through the downregulation of ADAM12 by HIF-1 activation. (**A**–**E**) Results for the RNA-seq of UV-F2 cells stably overexpressing T-cadherin (F2T cells). F2T cells cultured in DMEM containing 5% serum from adiponectin knockout mice were treated with or without roxadustat (Roxa) (50 μM) in the presence of high molecular weight adiponectin (HMW-APN) (10 μg/mL) for 24 h (n = 3 for each group). (**A**) Principal component analysis of the gene expression profile. (**B**) The number of differentially expressed genes. The false discovery rate (FDR) was set to 0.1 and the number of genes with twofold or more change was noted. The color scale shows the processed log2-fold change in fragments per kilobase per million (FPKM), representing the mRNA expression of each gene in green (low)-black-red (high). (**C**) Upregulated genes of gene ontology (GO) cellular component analysis. (**D**) Upregulated genes of the Kyoto Encyclopedia of Genes and Genomes (KEGG) pathway analysis. Data show regulations in roxadustat/adiponectin compared to adiponectin. (**E**) Heatmap for mRNA expression levels associated with focal adhesion and ECM-receptor interaction in KEGG pathway analysis. The color scale shows processed the log2-fold change in FPKM, representing the mRNA expression of each gene in green (low)-yellow–red (high). (**F**) Quantitative PCR analysis of ADAM12 mRNA. F2T cells were treated with or without roxadustat (50 μM) for 24 h (n = 3 for each group). Data are means ± SEMs. ***p < 0.001 (unpaired t test). (**G**) Western blot analysis of total cell lysates. UV-F2 cells transfected control (Cont) or *Adam12* siRNA were treated with or without roxadustat (50 μM) for 48 h (n = 3 for each group). (**H**) Western blot analysis of EVs isolated from cell culture medium by differential ultracentrifugation. In the presence of HMW-APN (20 μg/mL), UV-F2 cells transfected control or *Adam12* siRNA were cultured in FBS-free Advanced DMEM with or without roxadustat (50 μM) for 48 h (n = 3 for each group). Alix, TSG101, and syntenin were evaluated as EV markers. Data are means ± SEMs. *p < 0.05, **p < 0.01, and ***p < 0.001 (Tukey–Kramer test).

We further explored the T-cadherin stabilizing mechanism of roxadustat in the presence of adiponectin by RNA-seq analysis using F2T cells. Roxadustat dramatically changed gene expression, as shown in principal component analysis (Fig. 4A), with 955 upregulated differentially expressed genes (DEGs) and 734 downregulated DEGs (Fig. 4B). Interestingly, gene ontology (GO) cellular component analysis showed that focal adhesion and endosome/intracellular membrane dynamics were upregulated (Fig. 4C), while Kyoto Encyclopedia of Genes and Genomes (KEGG) pathway analysis suggested the well-known HIF-1 dependent pathways were upregulated (Fig. 4D). T-cadherin is a GPI-anchored membrane protein, and thus the stability of this protein may rely on the presence of neighboring proteins residing on the plasma membrane. Therefore, we focused on membrane proteins and found that roxadustat significantly increased the syndecan-4 gene (*Sdc4*) (Fig. 4E and Supple. Fig. 2A), a family member of syndecan, which increases EV biogenesis by activating the syntenin/Alix pathway^20^. However, contrary to our supposition, overexpression of syndecan-4 did not affect T-cadherin protein expression (Supple. Fig. 2B), and knockdown of the syndecan-4 gene further increased T-cadherin protein (Supple. Fig. 2C). Syndecan-4 was reported to interact with ADAM12 at the plasma membrane and offers a scaffold for this proteinase^21^. ADAM family metalloproteinases are known to shed various cadherins at their juxta membrane sites^22–26^. Among ADAM-family members, knockdown of the ADAM12 and ADAM15 genes increased T-cadherin protein levels (Supple. Fig. 2D), and both RNA-seq and quantitative PCR data revealed that *Adam12*, but not *Adam15*, was downregulated by roxadustat (Fig. 4E and F). Indeed, the knockdown of ADAM12, even in the presence of roxadustat, significantly increased T-cadherin protein levels (Fig. 4G) and increased adiponectin-induced EV production (Fig. 4H). Therefore, the decreased expression of ADAM12 despite increased syndecan-4 may mediate enhanced T-cadherin protein levels by roxadustat.

### HIF-1 activator increased plasma EV numbers in wild-type but not in T-cadherin-deficient mice

**Figure 5:**
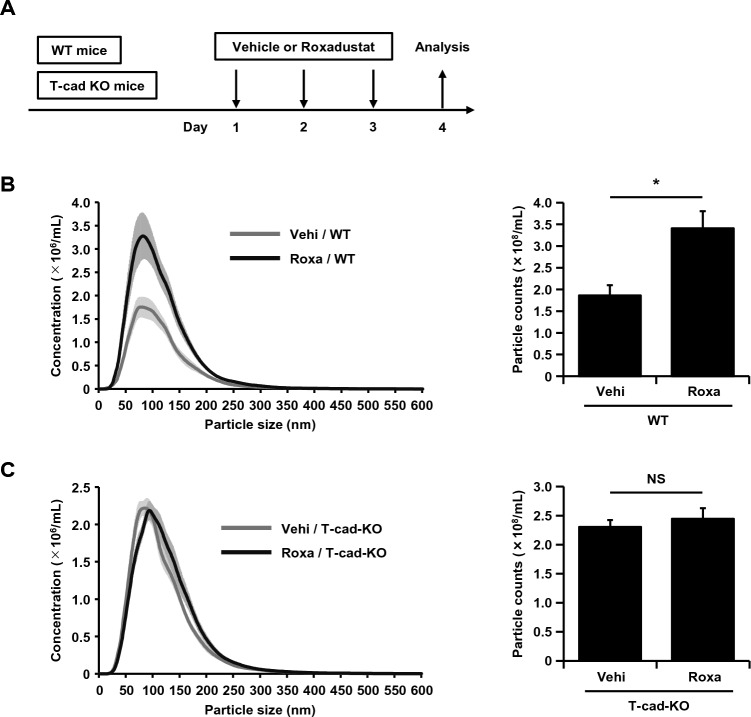
HIF-1 activator increased plasma EV numbers in wild-type but not in T-cadherin-deficient mice. (**A**) The experimental protocol. Vehicle (Vehi, 1% methylcellulose) or roxadustat (Roxa) (200 mg/kg/day) was orally administered to wild-type (WT) or T-cadherin knockout (T-cad KO) mice for three consecutive days and analyzed 24 h after the last dose (n = 6 for each group). (**B**) and (**C**) Particle numbers of plasma EVs analyzed by nanoparticle tracking analysis (NTA) of WT mice (**B**) and T-cad KO mice (**C**) treated with vehicle or roxadustat. Data are means ± SEMs. *p < 0.05 (unpaired t test). NS; not significant.

Finally, we tested whether roxadustat administration in mice increases the number of plasma EVs in vivo (Fig. 5A). To isolate EVs from mouse plasma, we used a Tim4 affinity purification method with magnetic capture of phosphatidylserine (PS). This system takes advantage of the property of the Tim4 protein to specifically bind PS displayed on the surface of EVs, allowing much lower contamination by non-EV proteins such as lipoproteins and aggregated proteins^27^. Three consecutive daily doses of roxadustat (200 mg/kg/day) in wild-type mice approximately doubled the number of purified plasma EVs as measured by nanoparticle tracking analysis (NTA) (Fig. 5B). Importantly, the same dose of roxadustat administered to T-cadherin-deficient mice did not alter plasma EV numbers (Fig. 5C).

## Discussion

Our study demonstrated that pharmacological HIF-1 activation increased T-cadherin mRNA levels and protein expression in cultured endothelial cells. Pharmacological HIF-1 activation also strongly increased EV production from the cells in the presence of adiponectin. Such increased EV production was abolished by silencing T-cadherin even in the presence of adiponectin, suggesting the dependence of this effect of EV on the adiponectin/T-cadherin system. The increased circulating EV numbers by roxadustat administration were observed in wild-type mice but not in T-cadherin-deficient mice.

The increase in the T-cadherin promoter activity by roxadustat in a reporter assay was statistically significant but small. Such a smaller induction of luciferase expression in HEK293 cells than the actual mRNA induction in endothelial cells by roxadustat may suggest the involvement of the indirect transcriptional regulation by HIF activation, in addition to the direct transactivation by HIF-1 itself. On the other hand, roxadustat did not alter T-cadherin mRNA stability after transcriptional inhibition by actinomycin D treatment of UV-F2 cells. Notably, this experiment also demonstrated the higher stability of T-cadherin mRNA than *Vegf* mRNA. Such high stability with small but statistically significant trans-induction of T-cadherin mRNA may partly explain why T-cadherin gene expression was gradually increased by HIF activators compared to representative HIF-responsive genes such as *Vegf* and *Gapdh*.

Overactivation of HIF-1 by pharmacological activators but not by hypoxia treatments upregulated the protein level of T-cadherin. Hypoxic conditions induce not only HIF-1 activation but also the ER stress response^28,29^. We recently reported that activation of inositol-requiring enzyme 1 alpha (IRE1α), the canonical ER stress signaling pathway, attenuated both T-cadherin expression and EV production^17^. Therefore, under hypoxic conditions, the ER stress response-induced downregulation of T-cadherin may override the HIF-1-mediated increase in T-cadherin expression. Increased expression of the ER stress marker C/EBP homologous protein (Chop) was observed in cells treated with higher doses of roxadustat and dose-dependent T-cadherin expression was attenuated, suggesting that ER stress may override HIF-1 activation-dependent induction of T-cadherin expression. Along with these notions, it would be impractical to speculate that T-cadherin expression is increased by hypoxia in vivo under ischemic conditions, such as myocardial infarction and acute kidney injury, both of which require adiponectin for organ protection^6,30^. Rather, our study demonstrated that pharmacological activation of HIF-1 resulted in upregulation of T-cadherin and EV production through the adiponectin/T-cadherin system.

T-cadherin exists in cells as 130 kDa with a prodomain and 100 kDa of mature forms^31^. We reported that the 130 kDa prodomain form has a higher affinity for adiponectin and a higher plasma membrane localization than the 100 kDa mature form^19^. HIF-1 activation preferentially upregulated the 130 kDa form over the 100 kDa form of T-cadherin, consistent with the enhancement of adiponectin accumulation and EV production^2,4^.

Interestingly, T-cadherin protein expression and EV production in the presence of adiponectin were significantly increased by roxadustat treatment even in cells stably overexpressing T-cadherin without a concomitant increase in its mRNA expression. When the cell surface-exposed proteins were biotinylated, roxadustat specifically attenuated degradation of biotinylated T-cadherin without affecting overall membrane protein turnover, suggesting specific regulation of protein stability. T-cadherin has been reported to interact with β1 and β3 integrins in neuronal cells^32^, and overexpression of T-cadherin increased β1 integrin in cultured cutaneous squamous cell carcinoma cells^33^. Although RNA-seq analysis showed upregulation of both β1 and β3 integrins with roxadustat, the knockdown of these integrins did not affect the abundance of T-cadherin protein in cultured endothelial cells (data not shown). Our RNA-seq analysis revealed potential effectors that may affect T-cadherin protein stability, such as syndecan-4 and ADAM12. Of these, expression of ADAM12 was downregulated by roxadustat, and knockdown of the ADAM12 gene significantly increased T-cadherin protein in the presence of adiponectin, demonstrating for the first time that this metalloproteinase destabilizes T-cadherin protein. ADAM family metalloproteinases are known to cleave various cadherins at their juxta-membrane sites^22–26^. Therefore, we tested whether ADAM12 could shed and reduce T-cadherin and found that knockdown of ADAM12 significantly increased both T-cadherin protein levels and synergistic EV production with adiponectin. Together with the transcriptional induction of T-cadherin mRNA by roxadustat, the attenuation of ADAM12 expression by roxadustat may mediate the upregulation of T-cadherin protein levels. A number of studies have demonstrated that T-cadherin is required to maintain cardiovascular homeostasis in pressure overload-induced cardiac hypertrophy, revascularization, and atherosclerosis models^30,34,35^. In our previous study, loss of ADAM12 in mice caused severe cardiac hypertrophy and dysfunction after transverse aortic constriction^36^. Therefore, the appropriate balance of gene expression changes by roxadustat may result in better outcomes being reported in various disease models using HIF-1 activators.

Our study showed that roxadustat administration significantly increased plasma EV levels in wild-type mice but not in T-cadherin-deficient mice. The increase in circulating EV levels observed with roxadustat in wild-type but not in T-cadherin-deficient mice may be due to the upregulation of T-cadherin expression and enhanced effects of adiponectin on EV production from T-cadherin-expressing cells, such as endothelial cells. We have previously demonstrated the T-cadherin-dependent effect of adiponectin on EV production using the same TIM4 affinity-based purification method used in this study^34,37^. We did not detect significant changes in T-cadherin mRNA or protein in the heart, one of the most abundant T-cadherin-expressing tissues in the body (data not shown). However, it is possible that T-cadherin expression was induced in limited cells in each tissue, such as vascular endothelial cells, in response to roxadustat, contributing to the significant increase in circulating EV levels. Further in vivo studies using tissue/cell-specific T-cadherin-deficient mice are needed to clarify the detailed role of T-cadherin in systemic EV regulation by HIF-1 activators. Although we have demonstrated the role of ADAM12 in T-cadherin protein stability in cultured endothelial cells, we have not clarified the relevance of this mechanism in other cell types or in vivo using ADAM12-deficient mice.

To date, several HIF-1 activators have been developed and reached clinical stages^38^. Interestingly, they have been reported to have potential not only in renal anemia but also in various diseases such as atherosclerosis and hypertension^10,11,15,16^, in which adiponectin also plays a protective role^30,39,40^. It may be reasonable to speculate that the pleiotropic organ protection by pharmacological HIF-1 activators is partly due to the enhancement of T-cadherin and subsequent EV production by adiponectin.

## Methods

### Cell cultures and transfections

Mouse vascular endothelial UV-F2 cells obtained from RIKEN Cell Bank and HEK293 cells were maintained in Dulbecco’s modified Eagle’s medium (DMEM) with 10% fetal bovine serum (FBS), 100 U/mL penicillin, and 100 μg/mL streptomycin. UV-F2 cells stably overexpressing T-cadherin (F2T cells) were generated as described^4^. HUVECs were purchased from Lonza and maintained in HuMedia-EG2 (Kurabo) up to passage 6. Human adipose tissue-derived mesenchymal stem cells (hAD-MSCs) were obtained from Lonza Ltd. and maintained in Mesenchymal Stem Cell Growth Medium 2 purchased from Promocell Gmbh induced by culture in collagen I-coated plates with DMEM supplemented with 2% horse serum.

For siRNA experiments, UV-F2 cells were transfected with Silencer Select Predesigned siRNA (Ambion), *Cdh13* (T-cadherin) (ID: s63759), *Sdc4* (s76467), and *Adam9*, *10*, *12* (s61952), *15*, *17*, and *19* by using Lipofectamine RNAiMAX Reagent (Invitrogen). For overexpression of Syndecan-4 in UV-F2 cells, the cDNA of mouse *Sdc4* was cloned and inserted into pLVSIN-CMV-puro lentiviral vector, and UV-F2 cells infected with the resultant lentivirus were selected in the presence of 2 μg/mL puromycin.

### Quantitative PCR

Sample mRNA was transcribed to cDNA by using ReverTra Ace™ qPCR RT Master Mix (TOYOBO), and quantitative PCR was performed by PowerUp SYBR Green™ Master Mix (Applied Biosystems) using a QuantStudio7 Real-Time PCR System (Applied Biosystems). Each result was normalized to the *18s* mRNA expression level.

The following primers we used for quantitative PCR analysis; mouse *18s*: CGGCTACCACATCCAAGGAA (forward) and GGTCCTCGATGAATTCGGCA (reverse), human *18s*: GGCCCTGTAATTGGAATGAGTC (forward) and CCAAGATCCAACTACGAGCTT (reverse), mouse *T-cadherin*: AAGATGCAGCCGAGAACTCC (forward) and GGTCCTCGATGAATTCGGCA (reverse), human *T-cadherin*: GGCCCTGTAATTGGAATGAGTC (forward) and CCAAGATCCAACTACGAGCTT (reverse), mouse *Gapdh*: GCACAGTCAAGGCCGAGAAT (forward) and GCCTTCTCCATGGTGGTGAA (reverse), human *Gapdh*: AAGGGCATCCTGGGCTACA (forward) and GAGGAGTGGGTGTCGCTGTTG (reverse), mouse *Vegfa*: GATCAAACCTCACCAAAGCC (forward) and TCTTTCTTTGGTCTGCATTCAC (reverse), human *Vegfa*: CAGAATCATCACGAAGTGGTG (forward) and GAAGATGTCCACCAGGGTC (reverse), mouse *Chop*: CTGGAAGCCTGGTATGAGGAT (forward) and CAGGGTCAAGAGTAGTGAAGGT (reverse), human *Chop*: CATGTTAAAGATGAGCGGGTG (forward) and CACTTCCTTCTTGAACACTCTC (reverse), mouse *Syndecan-4*: AGATCTGGATGACACGGAG (forward) and TCAGGGATGTGGTTATCCAG (reverse), and mouse *Adam12*: ACGTACAGCTTAGAGCCAATGA (forward) and CCCGCATTTGAGAGGTTCCA (reverse).

### Reporter gene assay with the T-cadherin promoter

The promoter region of the mouse *Cdh13* gene, 1 to 949 bp upstream of the translation initiation codon ATG, was cloned from C57J/B6 mouse cDNA, and inserted into the multicloning site of the pGL3-Basic vector (Promega). This vector and the pRL-CMV vector (Promega) were co-transfected into HEK293 cells using the Lipofectamine 2000 reagent (Invitrogen). Firefly and Renilla luciferase activities were measured using the Dual-Luciferase Reporter Assay System (Promega) and the Centro XS 3 LB960 microplate luminometer (Berthold Technologies). Firefly luciferase activity relative to Renilla luciferase activity, an internal standard, was used to quantify *cdh13* promoter activity.

### Isolation of EV

Culture supernatants of UV-F2 or F2T cells cultured in 1200 μL of Advanced DMEM (Gibco) without FBS for 48 h in a 6-well plate were centrifuged at 800×*g* for 10 min to remove floating cells and debris, respectively. For EV isolation, 1000 μL of these supernatants was ultracentrifuged as previously described^4^. The EV pellets were directly solubilized in Laemmli sample buffer and subjected to sodium dodecyl sulfate–polyacrylamide gel electrophoresis (SDS-PAGE) for quantification of EV markers.

### Western blotting

Isolated EVs or cell lysates were loaded onto e-PAGEL, 4–20% gradient SDS-PAGE gels (ATTO), and transferred to nitrocellulose membranes. The membranes were blocked by Blocking One (Nacalai Tesque) and incubated with primary antibodies using Can Get Signal Immunosignal Enhancer Solution 1 (TOYOBO) overnight at 4 ℃ and with secondary antibodies using Solution 2 for 60 min at room temperature. The top and bottom of the membranes were cut according to the size of the proteins of interest prior to hybridization with primary antibodies. Chemiluminescence signals were developed with Chemi-Lumi One Super (Nacalai Tesque), visualized by ChemiDoc Touch MP (Bio-Rad), and quantified using Image Lab 6.0.1 (Bio-Rad). Each result was normalized to the α-tubulin protein level.

The following primary antibodies were used: goat polyclonal anti-adiponectin (AF1119, R&D Systems); goat polyclonal anti-T-cadherin (AF3264, R&D Systems); rabbit monoclonal anti- GAPDH (2118S, Cell Signaling Technology); rabbit monoclonal anti-ALIX (ab186429, abcam); rabbit monoclonal anti-TSG101 (ab125011, Abcam); rabbit polyclonal anti-syntenin (ab19903, Abcam); and rabbit monoclonal anti-α-tubulin (2125S, Cell Signaling Technology).

The following secondary antibodies were used: horseradish peroxidase conjugated (HRP conjugated) donkey anti-goat IgG (HAF109, R&D Systems); and HRP conjugated donkey anti-rabbit IgG (NA934V, GE Healthcare).

### Immunoprecipitation

T-cadherin protein was immunoprecipitated to quantify the T-cadherin protein expression level in HUVECs. The biotinylated monoclonal antibody clone 11A against human T-cadherin^19^ was mixed with cell lysates for 3 h at 4 ℃. Then, Streptavidin Sepharose™ beads (GE Healthcare) were added to the mixture and rotated for 3 h at 4 ℃. After extensive washing, elution was carried out by adding 6% SDS-sample buffer containing 0.1 M DTT and heating for 5 min at 98 ℃.

### Cell surface biotinylation

In the presence of HMW-adiponectin (10 μg/mL), F2T cells treated with or without roxadustat (50 μM) for 40 h were surface biotinylated with 0.25 mg/mL EZ Link Sulfo-NHS-LC-LC-Biotin (Thermo Scientific) in D-PBS(+) for 30 min at 4 ℃. After further incubation with or without roxadustat for 0, 0.5, 1, 2, and 4 h, the cells were harvested, and the resultant cell lysates adjusted to the same protein concentration were immunoprecipitated with Streptavidin Sepharose™ beads (GE Healthcare) for 3 h at 4 ℃. The streptavidin beads were then collected using Ultrafree -MC -SV Centrifugal Filters Durapore™ PVDF 5.0 µm (Merck Millipore) and washed with cell lysis buffer and TBE (25 mM Tris–HCl pH 7.4, 150 mM NaCl, 1 mM EDTA), followed by SDS-PAGE.

### Immunofluorescence staining

After cell surface biotinylation as described above, cells cultured with or without roxadustat for 40 h were incubated with 0.3% Triton-X 100 in D-PBS(−) for 15 min for membrane permeabilization and were blocked with 3% FBS in D-PBS(−) for 60 min. T-cadherin in the cells was visualized with Alexa Flour 594 goat anti-rat IgG (Invitrogen), following incubation with goat polyclonal anti-T-cadherin (AF3264, R&D Systems) overnight at 4 ℃. For the detection of biotinylated proteins, the cells were incubated with streptavidin conjugated with Alexa Fluor 488 (Invitrogen) for 60 min at room temperature. Cell nuclei were stained with DAPI. Stained cells were observed under FLUOVIEW FV3000 (Olympus).

### RNA-sequences

Total RNA was purified by the miRNeasy Mini Kit (Qiagen), and the quality of total RNA was analyzed using the RNA6000 Pico™ Kit (Agilent). We confirmed that the RNA integrity number (RIN) of the samples was equal to or greater than 6.5. RNA samples were sequenced on HiSeq 2500 (Illumina). Principal component analysis, the number of differentially expressed genes analysis, and heatmap for mRNA expression levels analysis were performed using iDEP ver. 1.1 on web application. GO pathway analysis and KEGG pathway analysis were performed using BioJupies. In the number of differentially expressed genes analysis, the false discovery rate (FDR) was set to 0.1 and the number of genes with twofold or more change was noted.

### Purification of high molecular weight adiponectin

HMW-adiponectin was purified by a T-cadherin-Fc affinity column and a gel-filtration column from a pooled mouse serum as previously reported^19^.

### Animals

Male C57BL/6J WT mice were purchased from CLEA Japan (Tokyo, Japan). T-cadherin KO mice were generated as previously described^18^ and bred on a C57BL/6J background. Mice were housed in 22 ℃ at a 12-h light/12-h dark cycle (lights on from 8:00 A.M. to 8:00 P.M.). roxadusutat (MedChemExpress) dissolved in 1% methylcellulose was orally administered to wild-type or T-cadherin KO mice at 8 to 9 weeks of age at 200 mg/kg/day for three consecutive days and analyzed 24 h after the last dose. In all experiments, mice were anesthetized by intraperitoneal injection of a mixture of medetomidine (0.3 mg/kg), midazolam (4 mg/kg), and butorphanol (5 mg/kg) and euthanized by bilateral pneumothorax after blood collection from the inferior vena cava.

The experimental protocol was approved by the Ethics Review Committee for Animal Experimentation of Osaka University School of Medicine (Ethical approval ID 03-056). This study also conforms to the Guide for the Care and Use of Laboratory Animals published by the US National Institutes of Health and is reported in accordance with ARRIVE guidelines.

### Plasma EV isolation and analysis

Heparinized plasma obtained from the inferior vena cava of a mouse under anesthesia was centrifuged at 10,000× for 30 min, and 100 μL of supernatant was used for EV isolation with MagCapture™ Exosome Isolation Kit PS Ver. 2 (FUJIFILM Wako). The purified EVs were counted with NanoSight™ LM10 (Malvern Panalytical).

### Statistical analysis

Statistical analysis was performed using JMP Pro 17 (SAS Institute). Data are shown as the means ± SEMs. Differences between the two groups were analyzed by Student's t test. A one-way ANOVA followed by the Tukey–Kramer test or Dunnett's test (for comparison with the control group) was performed for multiple comparisons. P values less than 0.05 were considered significant.

### Supplementary Information

Supplementary Figures.

## Data Availability

RNA-seq datasets were deposited to NCBI under accession number GSE242647 (reviewers token: yzyjiwiuhhifned). The datasets generated during this study are available from the corresponding author upon reasonable request. We submitted all raw datasets except RNA-seq data to DRYAD with DOI 10.5061/dryad.c866t1gdq.
