# Supplementary material for: Pharmacological HIF-1 activation upregulates extracellular vesicle production synergistically with adiponectin through transcriptional induction and protein stabilization of T-cadherin
Source: Sci Rep. 2024 Feb 13;14:3620. doi: 10.1038/s41598-024-51935-6 (PMC10864391; doi:10.1038/s41598-024-51935-6)

Pharmacological HIF-1 activation upregulates extracellular vesicle production synergistically with adiponectin through transcriptional induction and protein stabilization of T-cadherin.

Kohei Fujii[1], Yuya Fujishima[1*], Shunbun Kita[1, 2*], Keitaro Kawada[1], Keita Fukuoka[1], Taka-aki Sakaue[1], Tomonori Okita[1], Emi Kawada-Horitani[1], Hirofumi Nagao[1, 3], Shiro Fukuda[1], Norikazu Maeda[1, 4], Hitoshi Nishizawa[1, 3], and Iichiro Shimomura[1]

[1] *Department of Metabolic Medicine, Graduate School of Medicine, Osaka University, 2-2, Yamada-oka, Suita, Osaka, 565-0871, Japan*

[2] *Department of Adipose Management, Graduate School of Medicine, Osaka University, 2-2, Yamada-oka, Suita, Osaka, 565-0871, Japan*

[3] *Department of Metabolism and Atherosclerosis, Graduate School of Medicine Osaka University, 2-2, Yamada-oka, Suita, Osaka, 565-0871, Japan*

[4] *Department of Endocrinology, Metabolism and Diabetes, Faculty of Medicine, Kindai University, 377-2, Ohno-higashi, Osaka-Sayama, Osaka, 589-8511, Japan*

* Corresponding author: Yuya Fujishima M.D., Ph.D. and Shunbun Kita, Ph.D., Department of Metabolic Medicine, Graduate School of Medicine, Osaka University, 2-2, Yamada-oka, Suita, Osaka, 565-0871, Japan

Telephone: +81-6-6879-3732. Fax: +81-6-6879-3739.

Email to Yuya Fujishima: y.fujishima@endmet.med.osaka-u.ac.jp. Email to Shunbun Kita: shunkita@endmet.med.osaka-u.ac.jp

Supplementary Figure Legends

Supplementary Figure 1. Effects of roxadustat and hypoxia on T-cadherin expression in T-cadherin-expressing cells.

(A) HIF-1 consensus sequences are located upstream of the translation initiation region of both the human and murine T-cadherin genes. (B) Quantitative PCR analysis of murine endothelial UV-F2 cells treated with or without roxadustat (Roxa) (50 μM) for the time indicated (n=3 for each group). Data are means ± SEMs. $^{*}p<0.05$, $^{**}p<0.01$, and $^{***}p<0.001$ (unpaired t test). (C) and (D) Quantitative PCR analysis of human adipose tissue-derived mesenchymal stem cells (hAD-MSCs) (C) and human umbilical vein endothelial cells (HUVECs) (D) treated with roxadustat for 24 hrs at the indicated concentrations (n=3 for each group). (E) Western blot analysis of total cell lysates. HUVECs were treated with roxadustat for 48 hrs at the indicated concentrations (n=3 for each group). (F) *Chop* mRNA expression levels in UV-F2 cells and HUVECs 24 hrs after roxadustat treatment at the indicated concentrations (n=3 for each group). Data are means ± SEMs. $^{*}p<0.05$, $^{**}p<0.01$, and $^{***}p<0.001$ versus control (untreated cells) (Dunnett's test). (G) Quantitative PCR analysis of UV-F2 cells cultured under 21% (normoxia) or 5% (hypoxia) oxygen for 24 hrs (n=3 for each group). (H) Western blot analysis of total cell lysates of UV-F2 cells cultured under 21% or 5% oxygen for 48 hrs (n=3 for each group). Data are means ± SEMs. $^{***}p<0.001$ (unpaired t test).

Supplementary Figure 2. Effects of syndecan-4 and ADAM families on T-cadherin protein expression.

(A) Quantitative PCR analysis of UV-F2 cells stably overexpressing T-cadherin (F2T cells) treated with roxadustat (Roxa) (50 μM) for 24 hrs (n=3 for each group). Data are means ± SEMs. $^{***}p<0.001$ (unpaired t test). (B) Western blot analysis of total cell lysates. UV-F2 cells and those

stably overexpressing syndecan-4 (*Sdc4*) UV-F2 cells were treated with or without roxadustat (50 μM) for 48 hrs (n=3 for each group). (C) Western blot analysis of total cell lysates. UV-F2 cells transfected control or *Sdc4* siRNA were treated with or without roxadustat (50 μM) for 48 hrs (n=3 for each group). Data are means ± SEMs. $^{*}p<0.05$ and $^{**}p<0.01$ (Tukey-Kramer test). (D) Western blot analysis of total cell lysates of UV-F2 cells transfected control or *Adam* 2, 9, 10, 15, 17, or 19 siRNA.

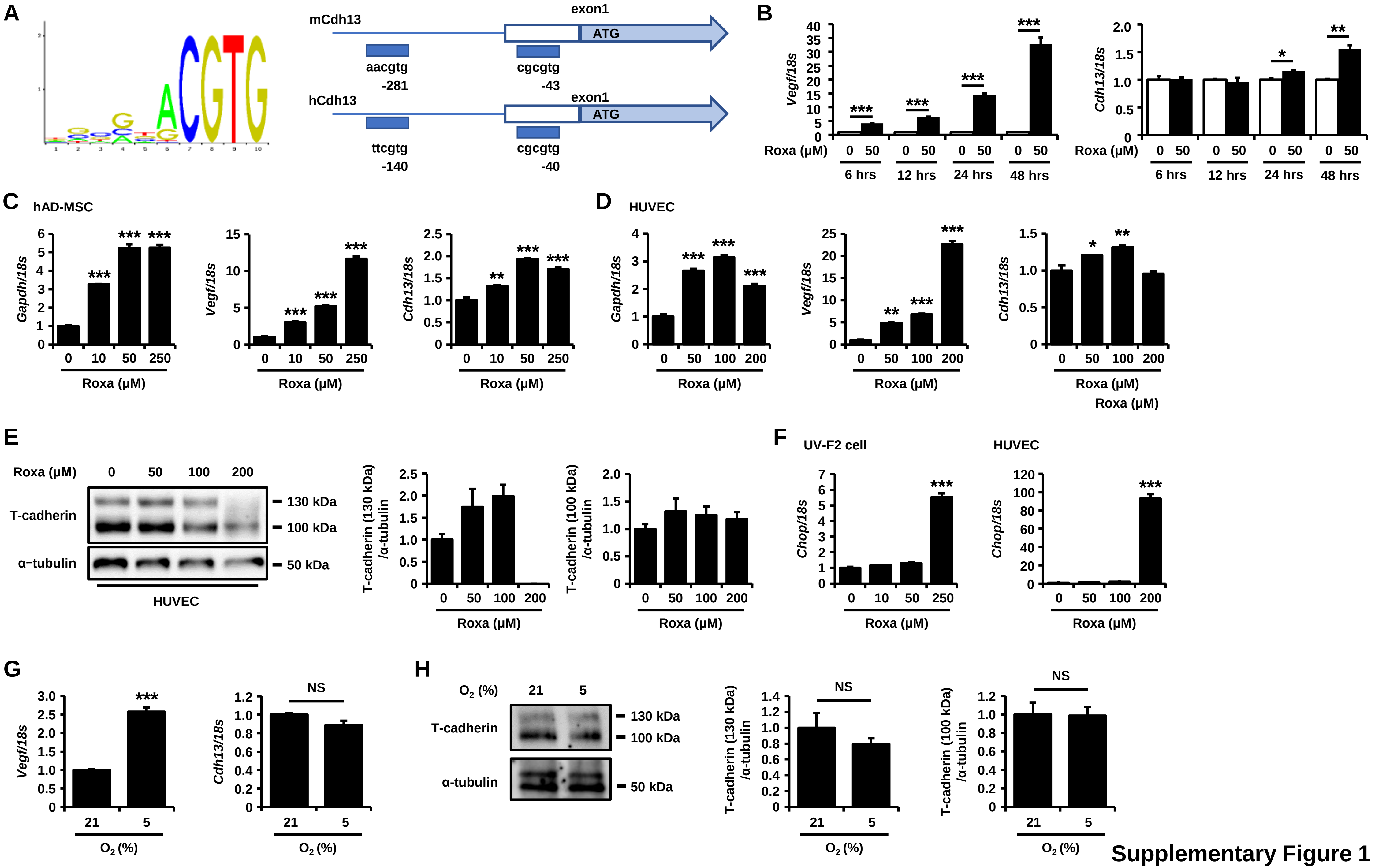

Supplementary Figure 1

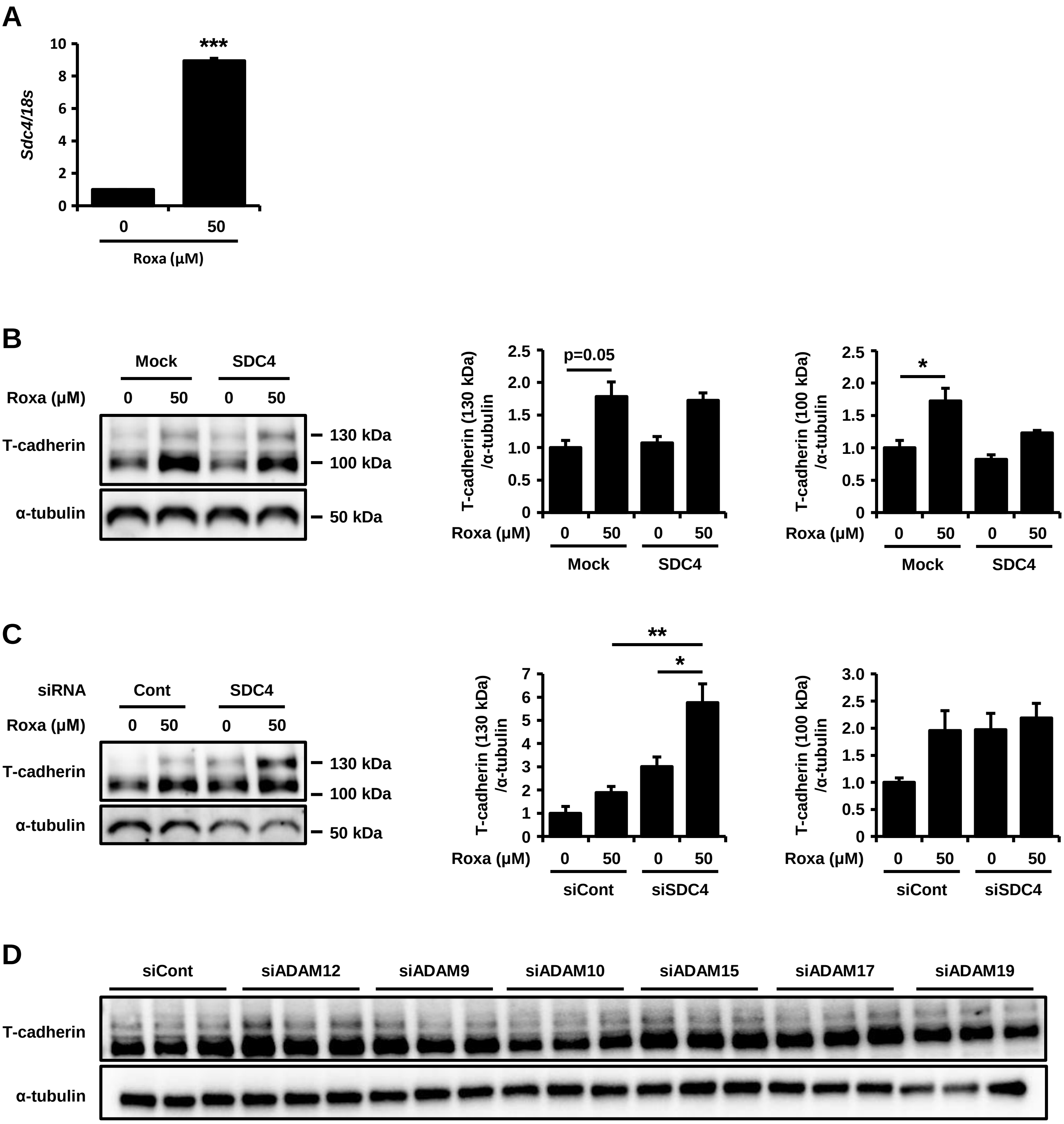

Supplementary Figure 2

Presentation of full-length gels and blots in Figures (Figures 1, 2, 3, and 4 and Supplementary Figures 1 and 2)

Figure 1E

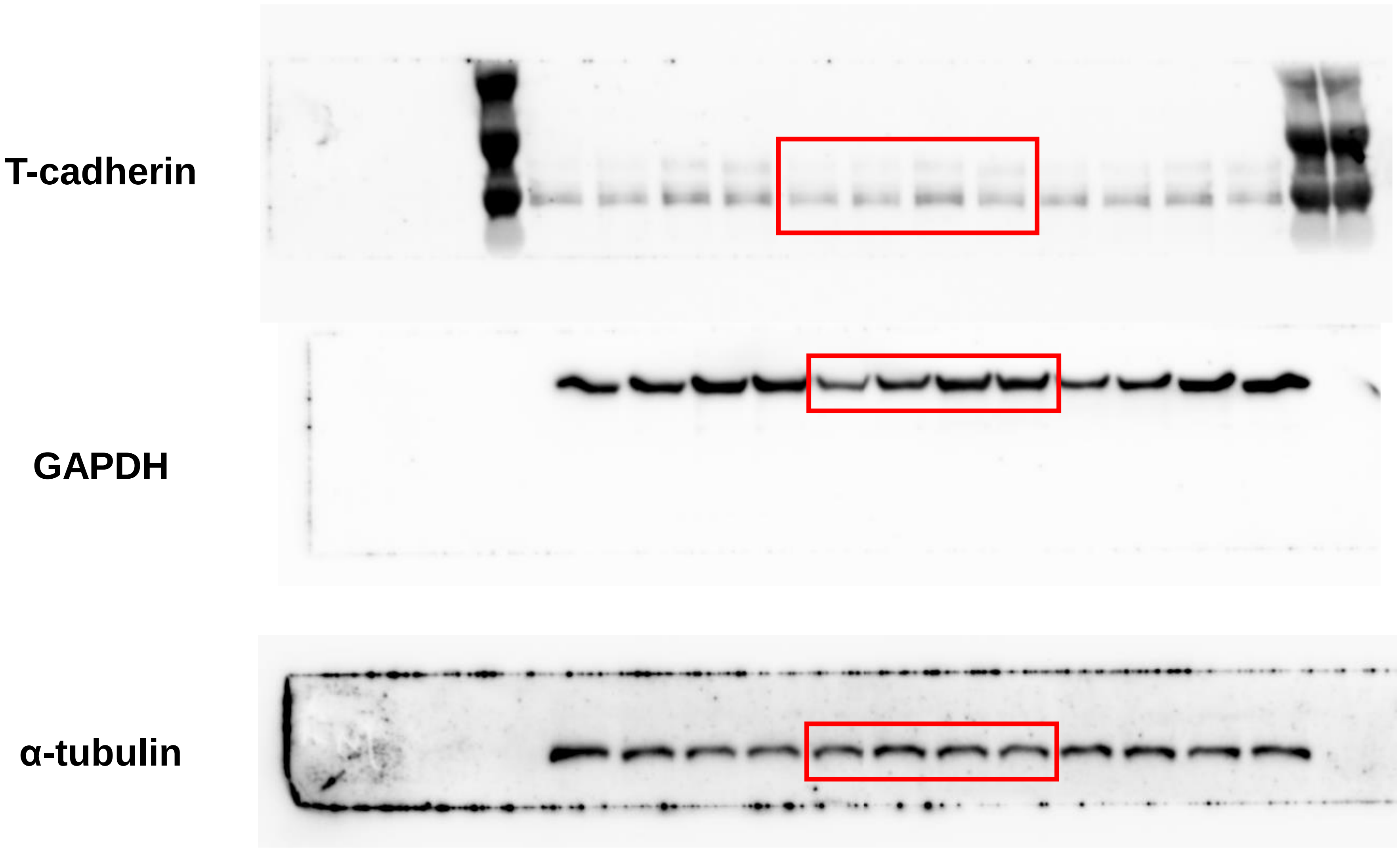

Figure 1F

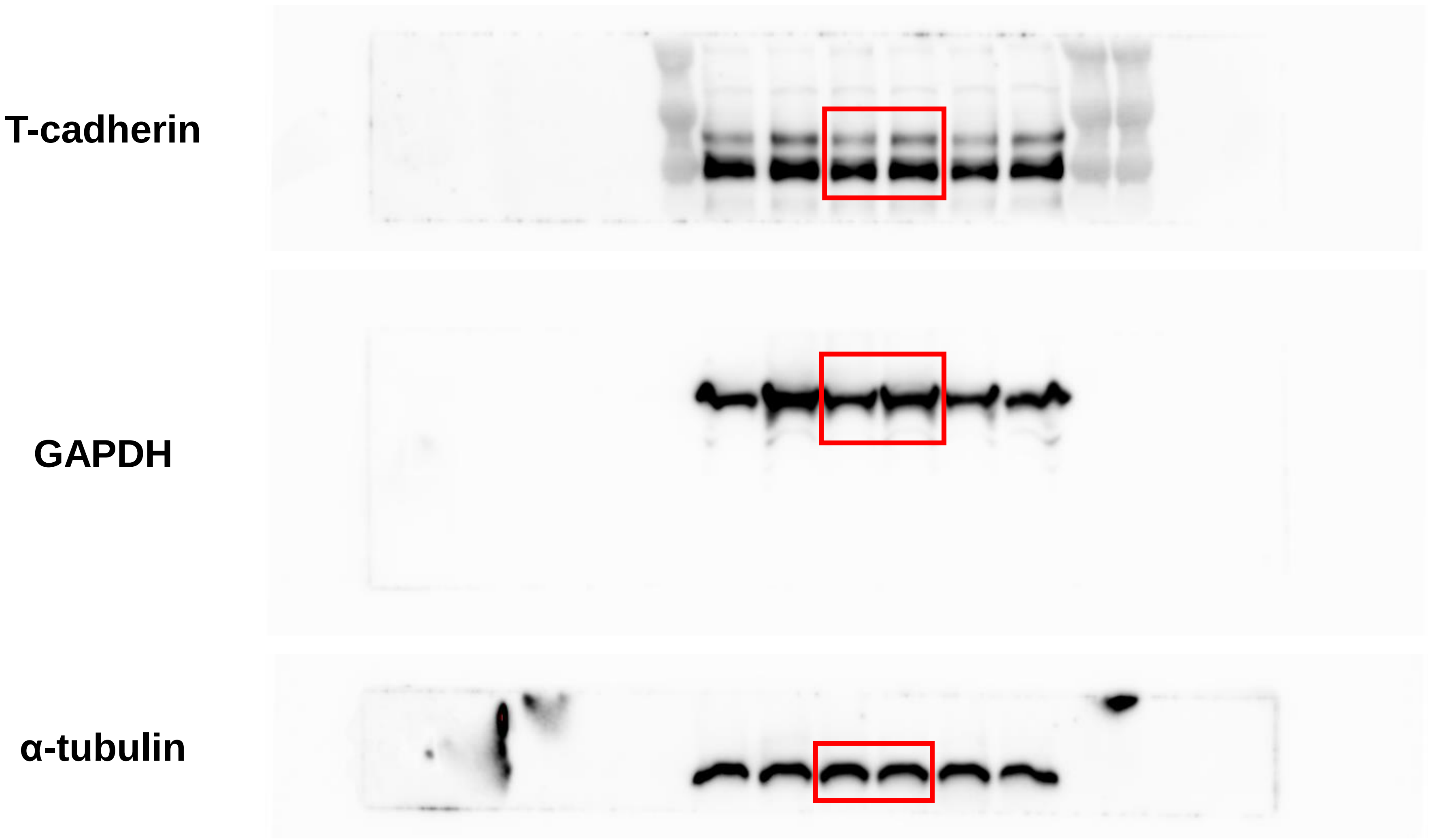

Figure 2A

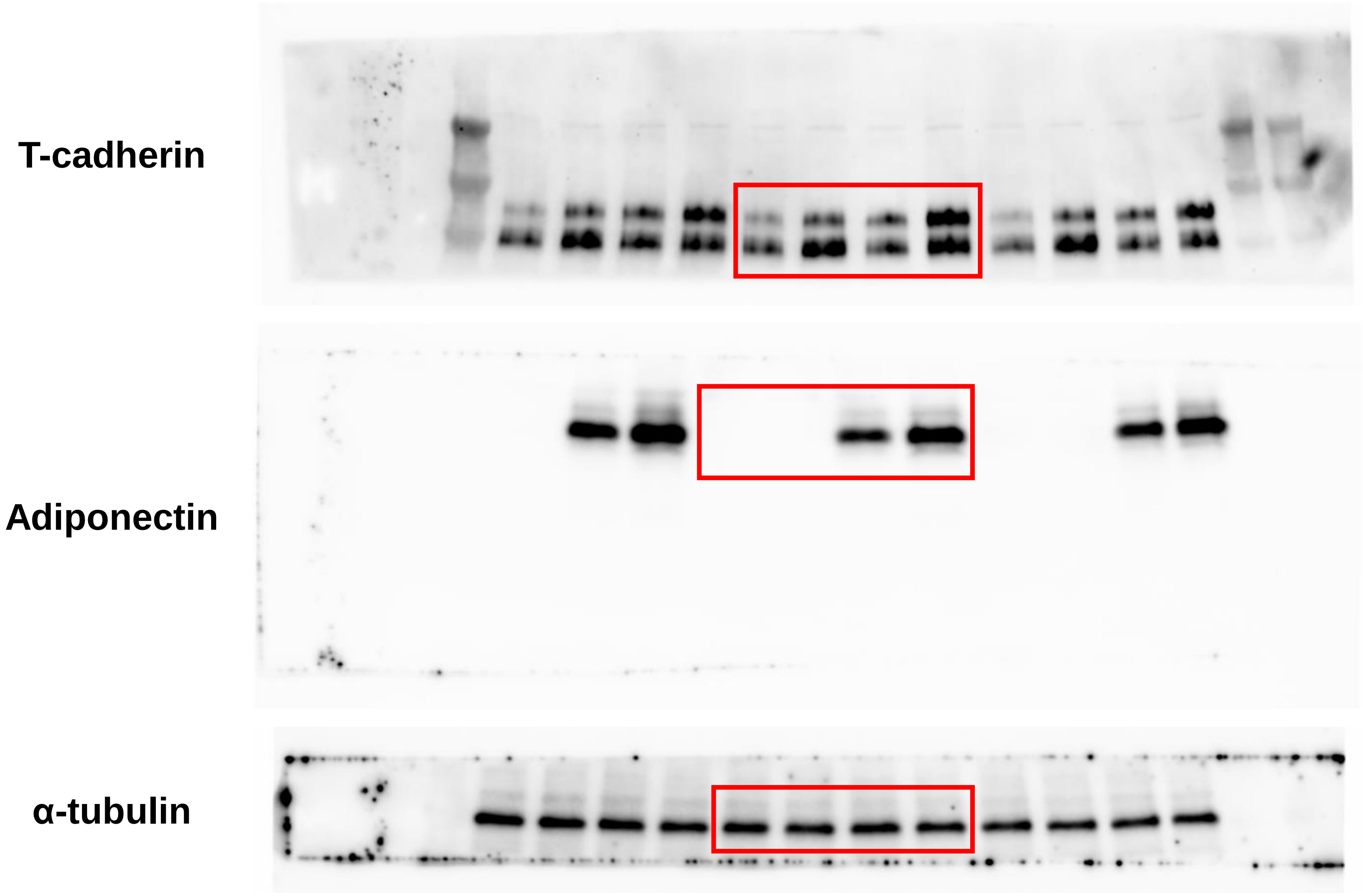

Figure 2B

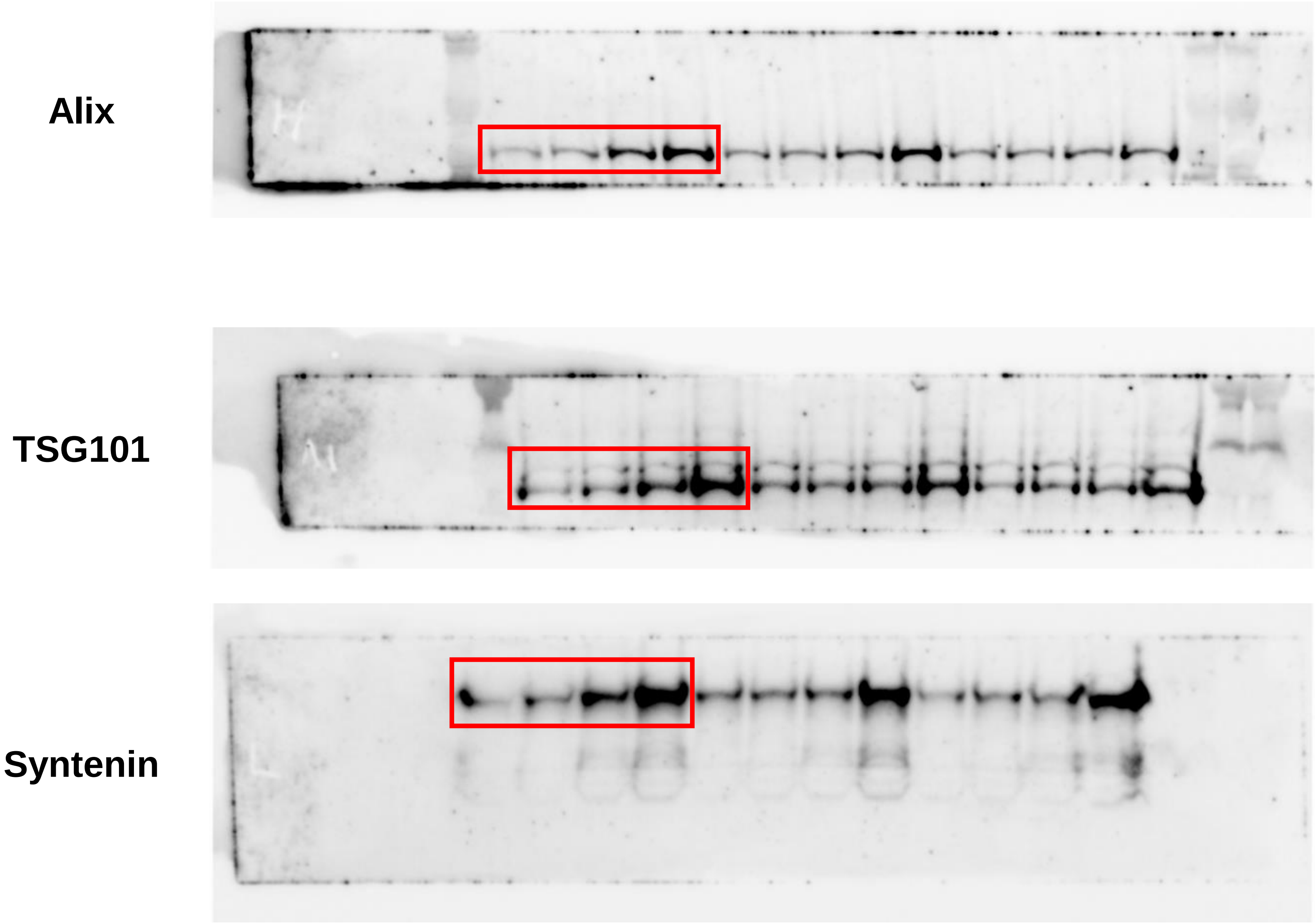

Figure 2C

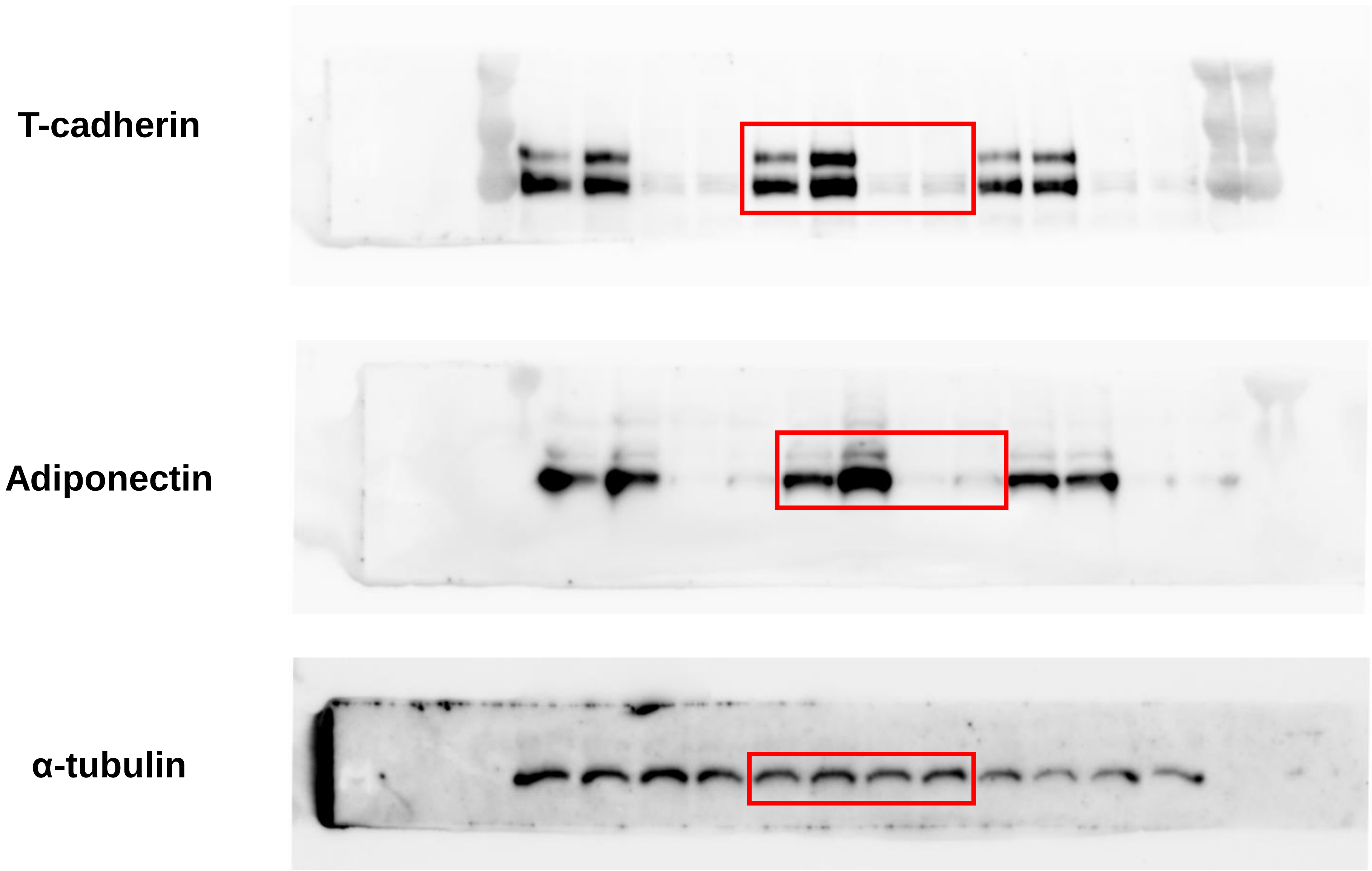

Figure 2D

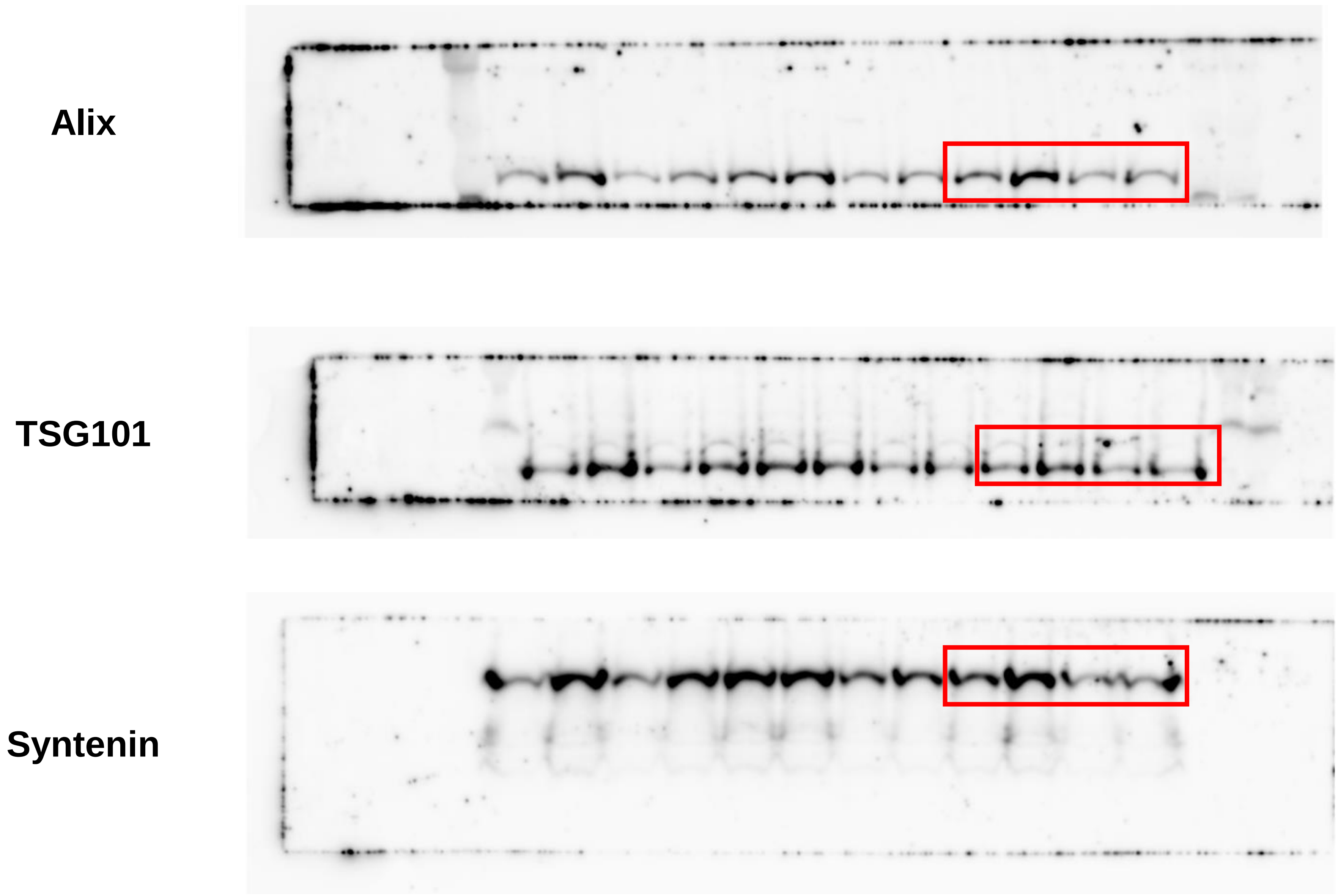

Figure 3B

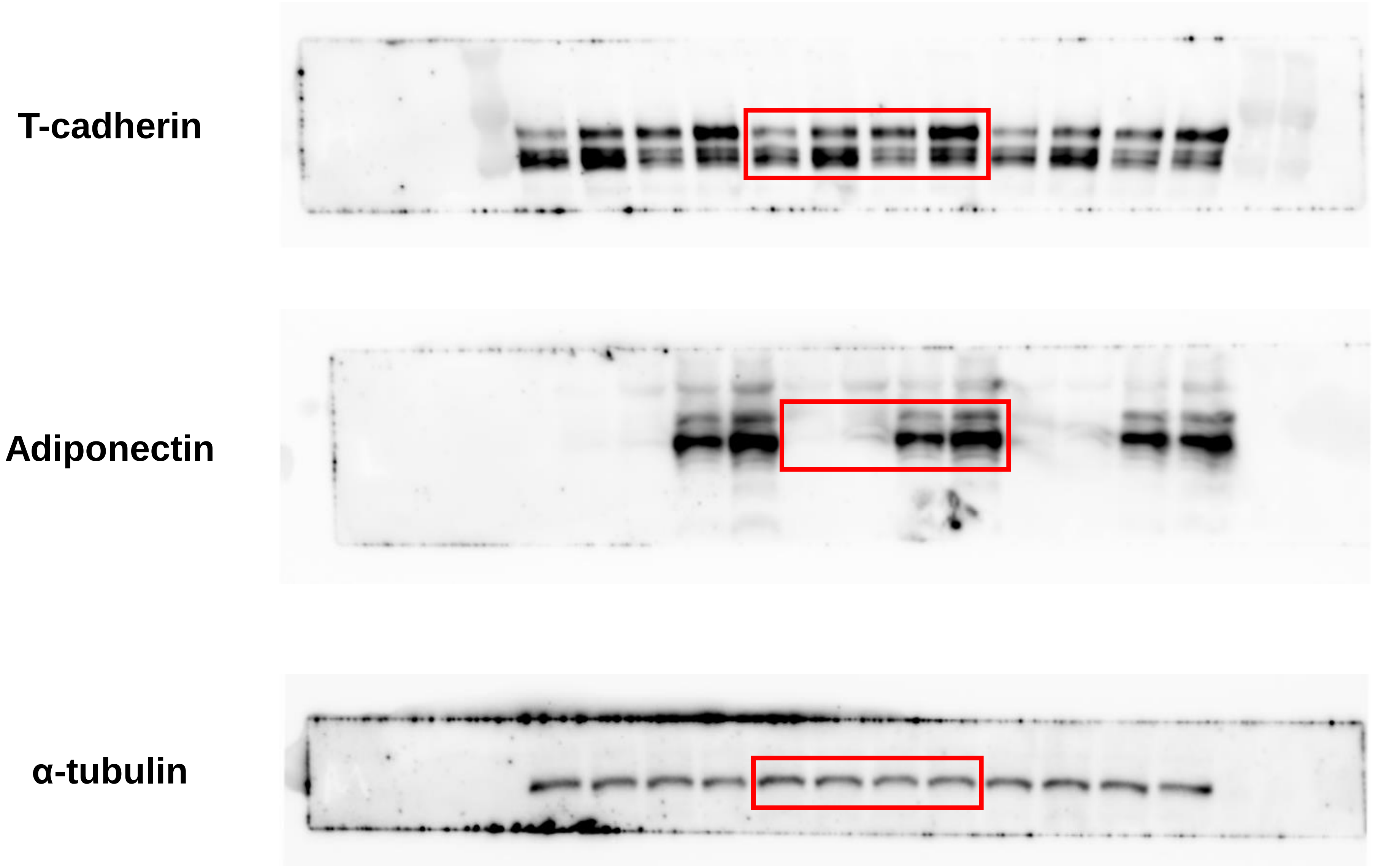

Figure 3D

T-cadherin
(Western blotting)

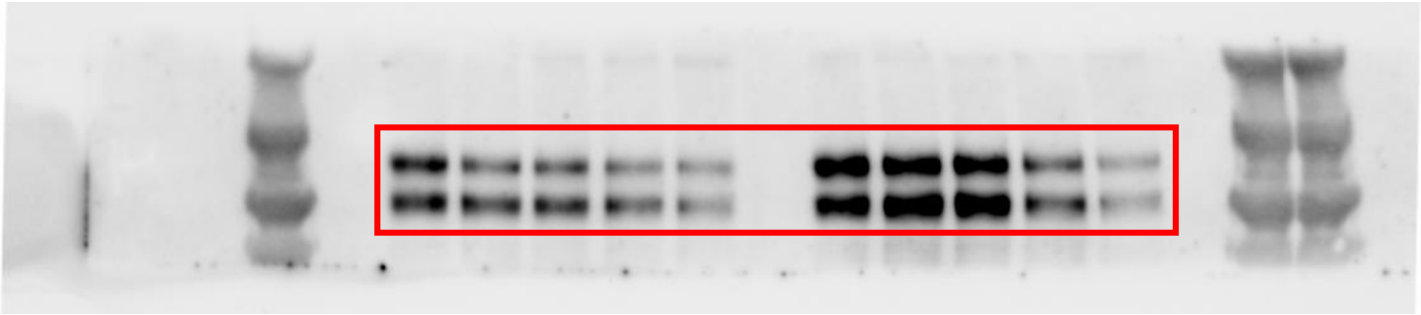

SYPRO Ruby
staining

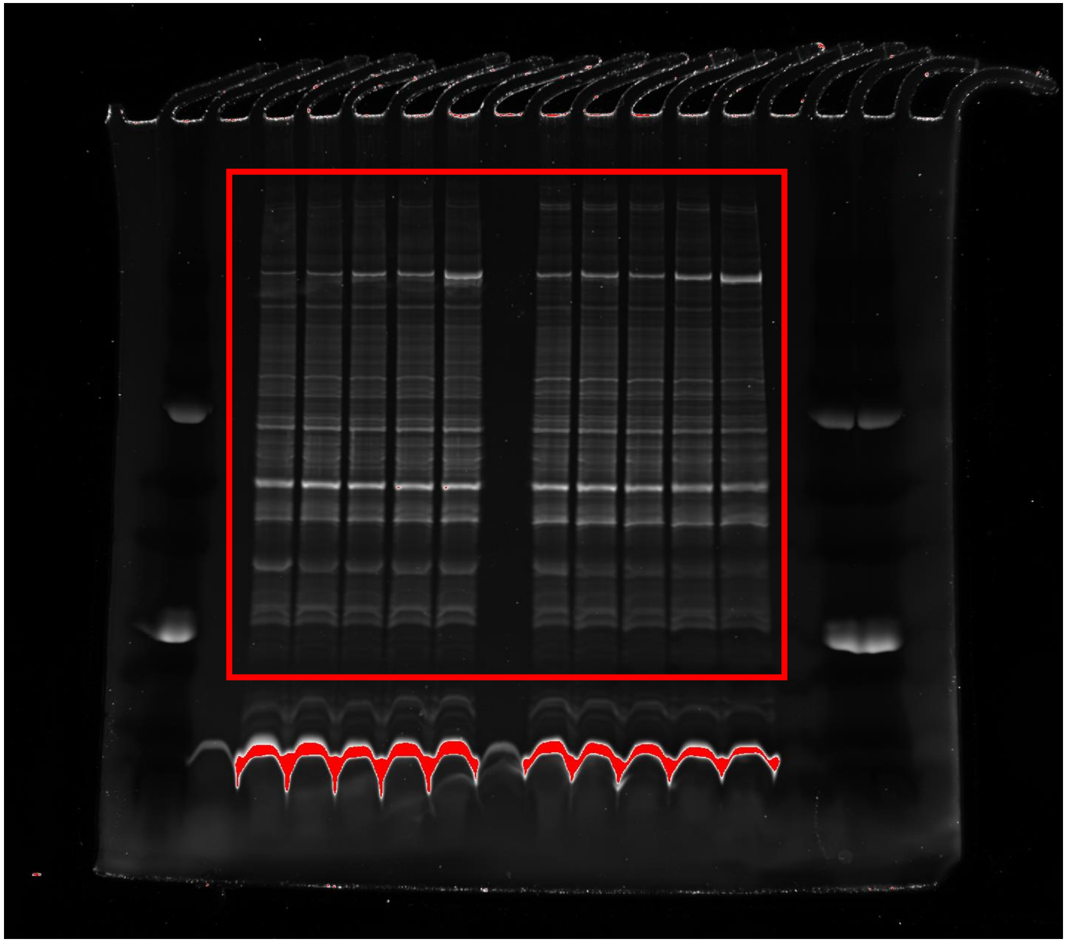

Figure 4G

T-cadherin

α-tubulin

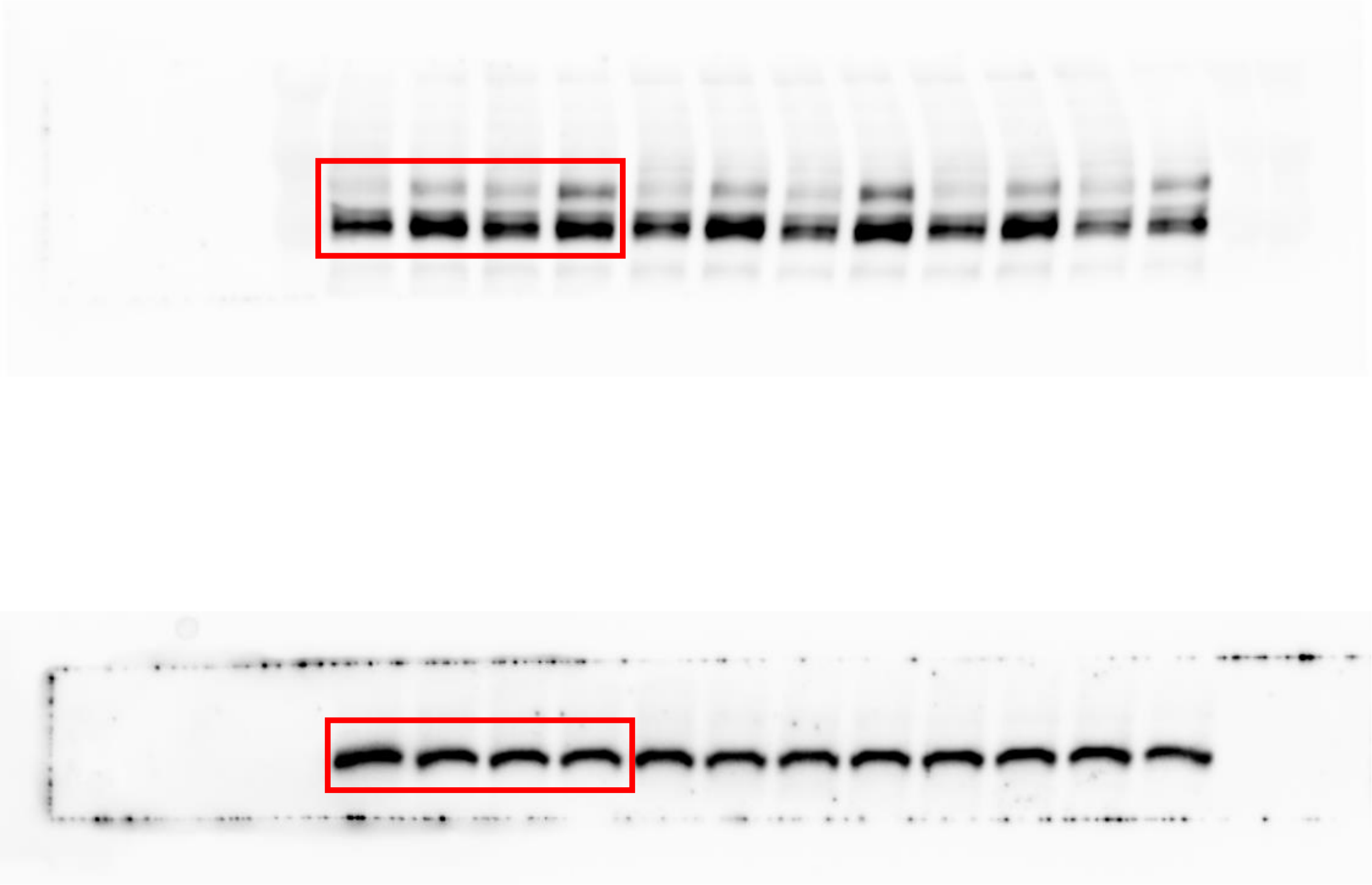

Figure 4H

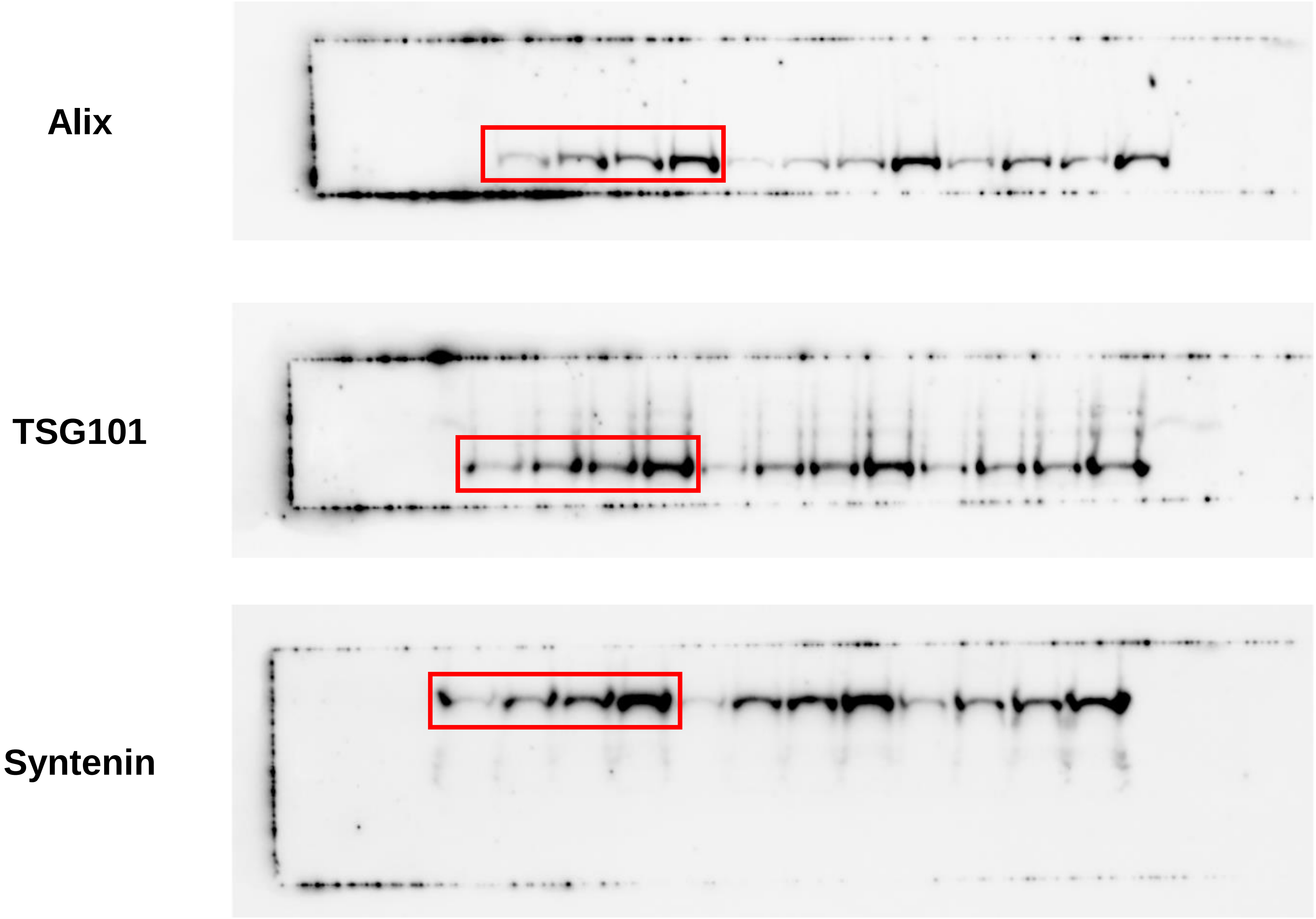

Supplementary Figure 1E

T-cadherin

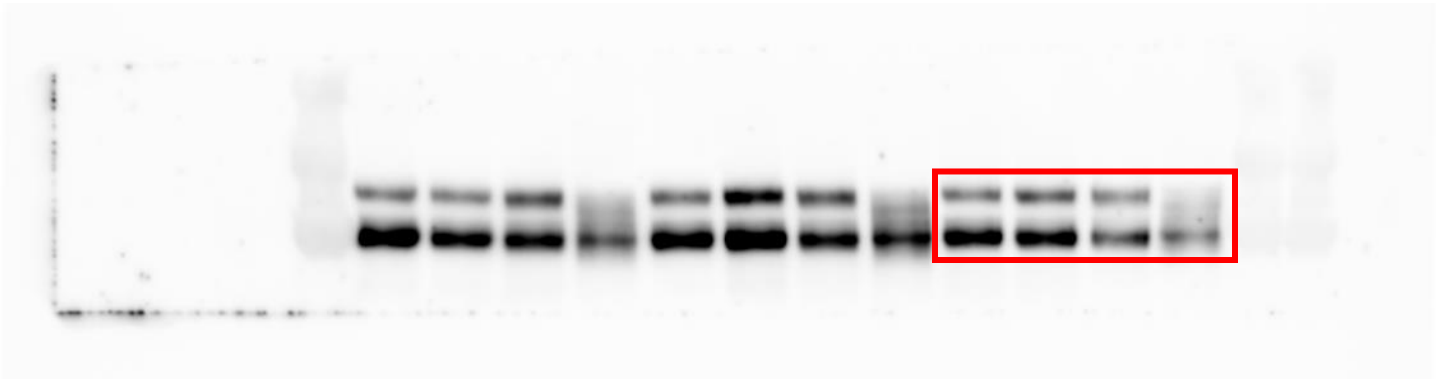

α-tubulin

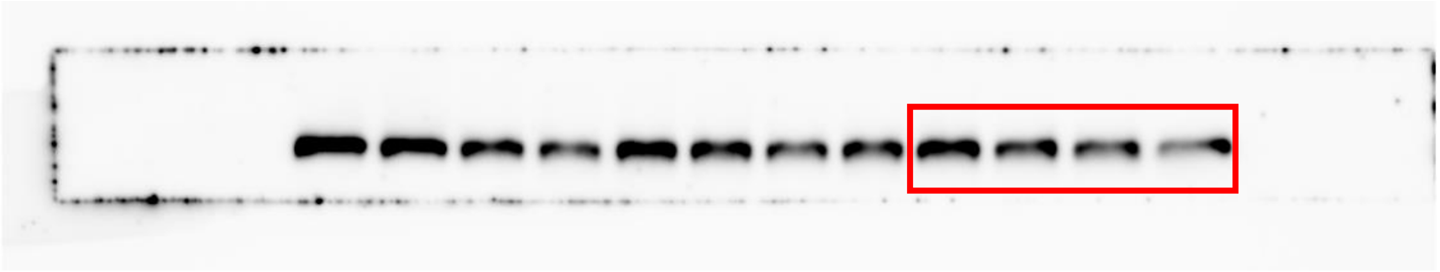

Supplementary Figure 1H

T-cadherin

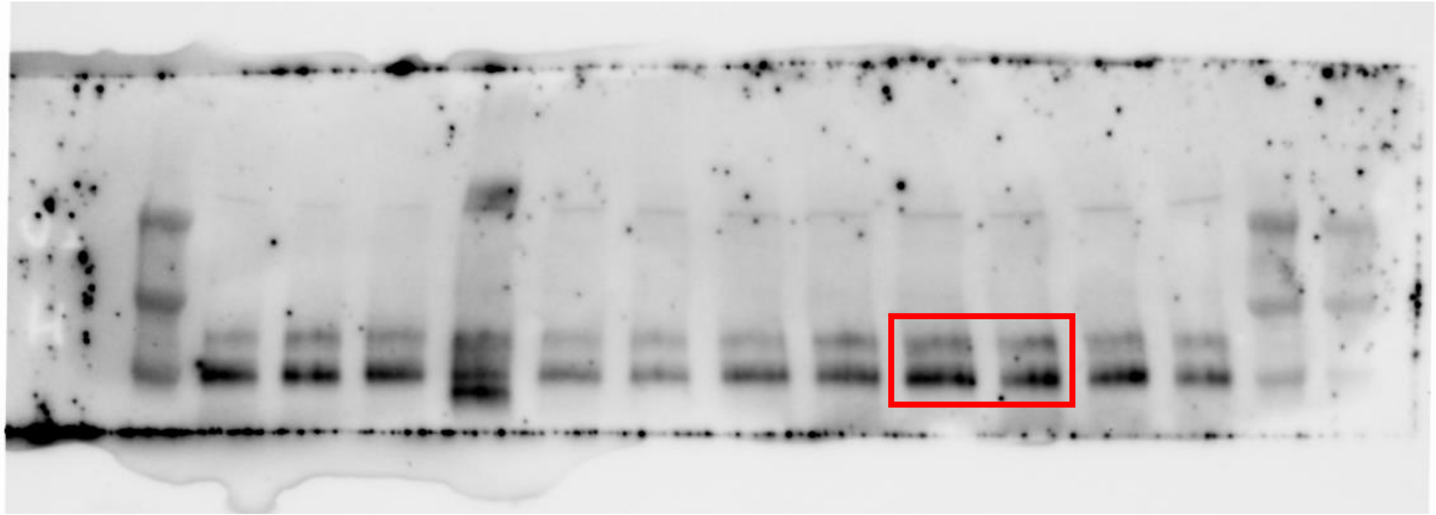

α-tubulin

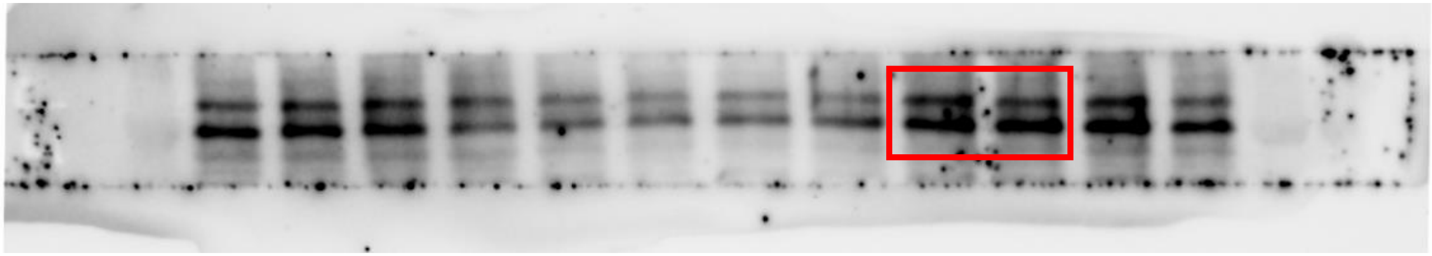

Supplementary Figure 2B

T-cadherin

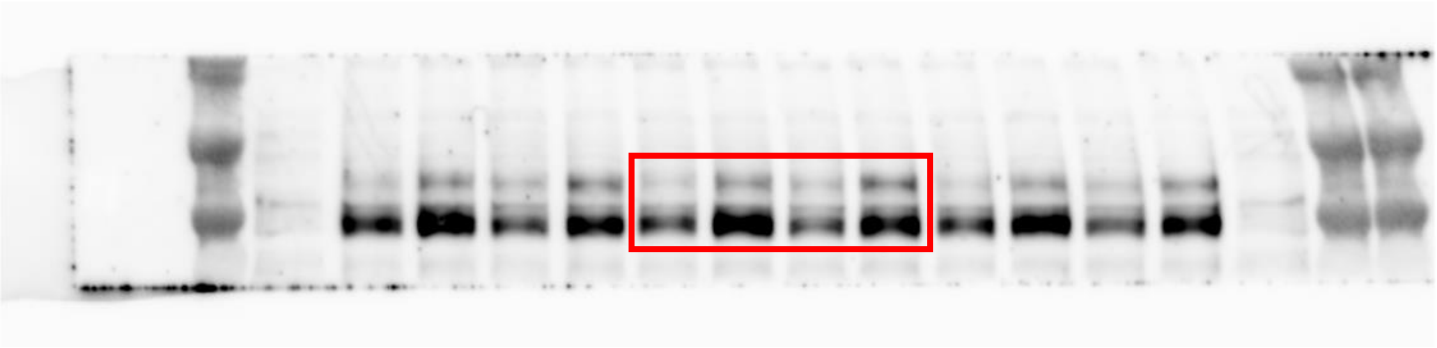

α-tubulin

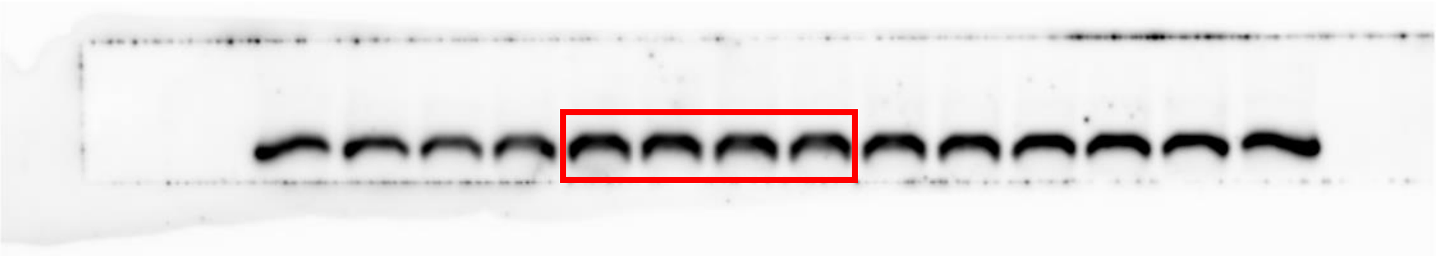

Supplementary Figure 2C

T-cadherin

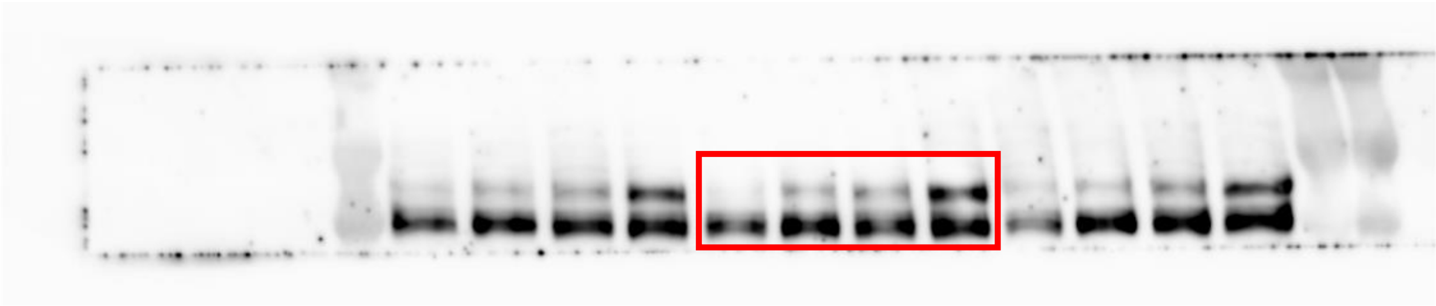

α-tubulin

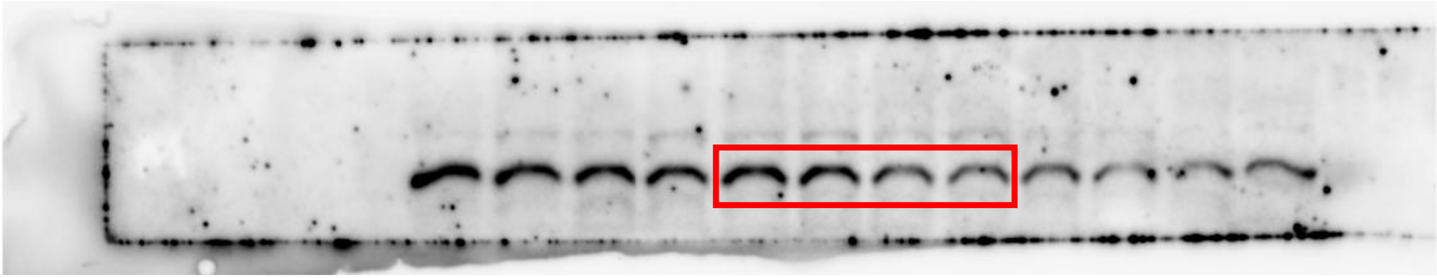

Supplementary Figure 2D

T-cadherin

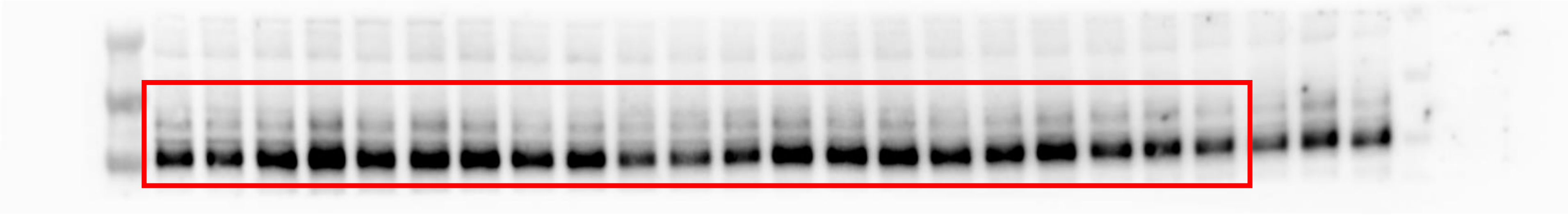

α-tubulin

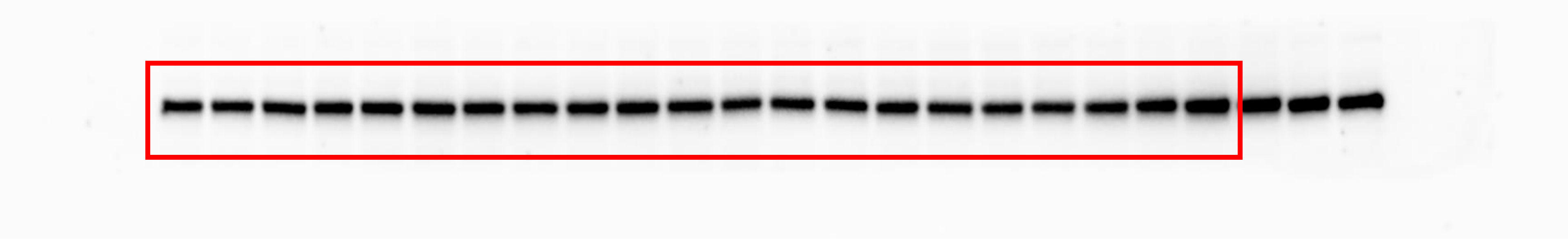

Supplement: Supplementary file 1 — Supplementary Figures. [file 41598_2024_51935_MOESM1_ESM.pdf]
